# Characterization of Bacteria Using Surface-Enhanced Raman Spectroscopy (SERS): Influence of Microbiological Factors on the SERS Spectra

**DOI:** 10.1021/acs.analchem.2c00817

**Published:** 2022-06-17

**Authors:** Danielle M. Allen, Gisli G. Einarsson, Michael M. Tunney, Steven E. J. Bell

**Affiliations:** † School of Pharmacy, Queen’s University Belfast, 97 Lisburn Road, Belfast, Northern Ireland BT9 7BL, UK; ‡ Centre for Experimental Medicine, School of Medicine, Dentistry and Biomedical Sciences, Queen’s University Belfast, 97 Lisburn Road, Belfast, Northern Ireland BT9 7BL, UK; § School of Chemistry and Chemical Engineering, Queen’s University Belfast, University Road, Belfast, Northern Ireland BT7 1NN, UK

## Abstract

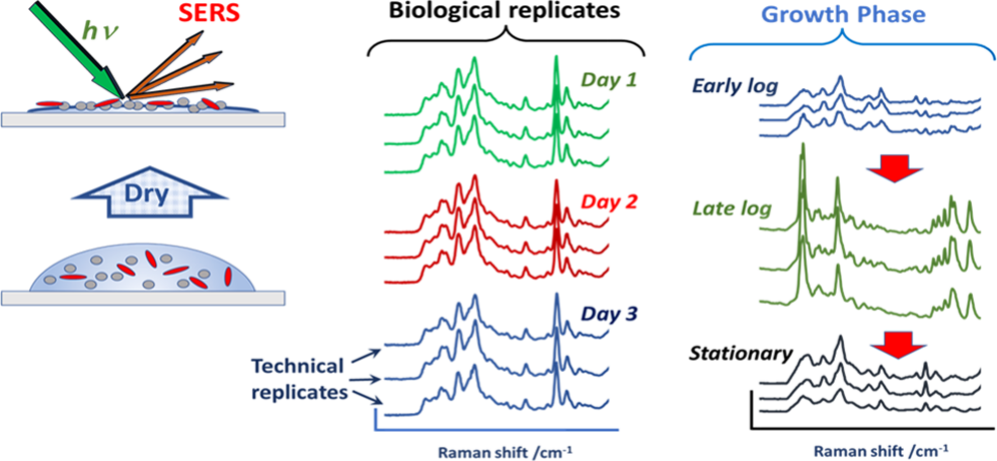

SERS is currently being explored as a rapid method for identification of bacteria but variation in the experimental procedures has resulted in considerable variation in the spectra reported for a range of bacterial species. Here, we show that mixing bacteria with a conventional citrate-reduced silver colloid (CRSC) and drying the resulting suspension yield highly reproducible spectra. These signals were due to intracellular components released when the structure of the bacteria was disrupted during sample preparation. This reproducibility allowed us to examine the effects of variables that do not arise in SERS of simple solutions but are relevant in studies of bacteria. These included growth phase and biological variation, which occurred when the same bacterial isolates were cultured under nominally identical conditions on different days. It was found that even under optimal standardized conditions the effect of differences in experimental parameters such as growth phase was very large in some bacterial species but insignificant in others. This suggests that it is important to avoid drawing general conclusions about bacterial SERS based on studies using small numbers of samples. Similarly, discrimination between bacterial species was straightforward when a small number of isolates with distinct spectral features were investigated; however, this became more challenging when more bacterial species were included, as this increased the possibility of finding different species of bacteria with similar spectra. These observations are important because they clearly delineate the challenges that will need to be addressed if SERS is to be used for clinical applications.

## Introduction

SERS has gained considerable attention as a potential method for the rapid identification of bacteria. Many studies have reported the characterization of a range of bacterial species, and some have even shown that it is possible to differentiate between different species using multivariate data analysis. Early studies were plagued with experimental problems; flavin adenine dinucleotide (FAD) dominated the spectra when excitation wavelengths below 514 nm were used.^1−5^ Culture media interference was another potential problem;^6^ however, it has been shown that the use of a stringent washing protocol removed all traces of media from the spectra.^7,8^ Later studies focused on sample preparation of the bacterial isolates for SERS measurements with two different methods dominating the literature. Simple mixing with a colloid is a convenient and rapid method, but it has been noted by some researchers that samples lacked uniformity and the resulting spectra were inconsistent and not reproducible.^5,9,10^ Many studies have therefore used *in situ* reduction, which involves impregnating or coating the bacterial cells with an enhancing material.^2,3,5,10−14^ It has been claimed that this method is superior as the close contact between the bacterial cells and the nanoparticles results in greater reproducibility.^5,10^ Unfortunately, an agreement between spectra from different research groups is typically poor, which is most likely due to differences in the experimental method followed. Some studies have focused on the effect on the spectra of different experimental parameters including excitation wavelength, laser power, exposure time of the sample to the laser, and the type of the substrate used.^15−18^ These have clearly demonstrated that these factors need to be controlled to obtain reproducible data even within a single research group.

There have been a reasonable number of studies investigating the effect of variations in the experimental parameters; however, the influence on the spectra of microbiological factors, including bacterial density, phase of growth, and the need for biological replicates, has not been well addressed. Some of these parameters would be expected to have a profound effect on the SERS spectra due to the associated differences in cell metabolism but may be controlled by standardizing experimental protocols. However, since bacteria are living organisms and do not necessarily respond or grow the same way on different days, there is also a need to understand the extent of day-to-day random variation that is intrinsic to biological samples and cannot be eliminated by adopting well-defined and understood experimental procedures. This means that in addition to recording technical replicates, which are defined as repeated measurements of the same sample, it is also important to include biological replicates, which are parallel measurements of distinct biological samples tested on different days. In the current study, we developed a standard experimental protocol, which had excellent reproducibility when tested using technical replicates. The optimized method was used for subsequent studies on biological replicates obtained from independent samples on different days; differences in the spectra were apparent and these were too large to be explained by experimental factors alone and therefore could be attributed to biological variation.

The overall picture that emerges from the relatively small (although carefully chosen) set of bacteria included in this study is that it is very difficult to make valid general conclusions about the SERS spectra of bacteria. Some species show very large spectral differences with the growth phase, while others do not; similarly, only some display significant spectral variation with biological replicates, while others have strongly conserved spectral features. This variation in the observed bacterial spectra means that it is possible to reach very different conclusions on, for example, the potential of SERS for discriminating between bacteria, depending on the samples chosen. In the current study, it was found that visualization of the results from principal component analysis (PCA) showed that even with just two principal components (PCs) it was possible to discriminate between five different bacterial species even when biological replicates, which increased the size of the clusters, were included. However, increasing the sample set to include further species led to an overlap of the clusters even when three PCs were included, making discrimination between some of the species more challenging. These observations are important in establishing what factors will be critical for the analysis of clinical samples, which may contain mixtures of several different bacterial species at different bacterial densities and phases of growth.

## Methods

### Sample Preparation

CRSC was prepared using a method adapted from that of Lee and Meisel.^19^ Bacterial isolates used in this study were selected from the Halo Research Group repository and stored at −80 °C until required. Bacterial isolates were grown overnight on agar plates, using the media and incubation conditions described in Table S1, and adjusted to an optical density (OD) of 0.15 before incubation. To test if the bacterial density (CFU/mL) affected the SERS spectra, samples were cultured after 6 h and adjusted to OD 0.1, 0.3, and 1.0. To calculate the CFU/mL, a five-fold serial dilution was performed in PBS at each OD, and colonies were counted at a dilution that gave between 10 and 30 colonies per 10 μL. To test if the phase of growth affected the SERS spectra, samples were cultured after 3, 6, and 24 h and the samples were adjusted to an OD of 0.3, to ensure a consistent bacterial density between samples. After incubation, 1 mL was centrifuged at 9000 rcf for 3 min, the bacterial pellet was washed 3 times with dH_2_O, and the supernatant was discarded. The bacterial pellet was mixed with 80 μL of CRSC and 10 μL bacterial colloidal aliquots were pipetted in triplicate onto a glass microscope slide covered in an aluminum foil and left to air dry. During the drying process, liquid evaporated from the edge of the droplet and was replenished with liquid from the interior, to create a coffee-ring, which was probed during the Raman measurement.

To determine if intracellular nucleotides were released in the supernatant when washed in water or mixed with CRSC, the bacterial pellet was obtained as described above but the supernatant was retained after each centrifugation step. To obtain the spectra, a 60 μL aliquot of the filtered supernatant (0.22 μm mesh filter) was mixed with 80 μL of CRSC; 10 μL aliquots of the mixture were then dried and analyzed. To determine if CRSC was causing bacterial cell death after simple mixing, bacterial pellets were mixed with either 80 μL of CRSC, CRSC supernatant, or PBS (growth control). At 0, 2, 4, 6, and 24 h, the CFU/mL was calculated as described above. To determine if drying the bacteria caused cell death, bacterial pellets were mixed with either 80 μL of CRSC or PBS and 10 μL aliquots were pipetted onto two 4 cm^2^ pieces of an autoclaved aluminum foil and left to air dry for 90 and 180 min. After drying, the aluminum foil was sonicated in 5 mL of PBS for 5, 10, and 20 min, and the CFU/cm^2^ was calculated as described above.

### Scanning Electron Microscopy (SEM)

The CRSC and bacterial samples were prepared in the same way as for SERS experiments, spotted onto SEM specimen stubs, and dried. A Quanta FEG 250 equipped with a field emission gun (FEG) was used. Samples were imaged with a 10 kV accelerating voltage and a spot size of 4.0.

### SERS Analysis

Measurements were carried out using a PerkinElmer RamanMicro 200 Raman spectrometer, which uses a 100 mW 785 nm external cavity diode laser and is based on an Olympus BX51 microscope chassis. The excitation light was focused onto the sample through a 20× objective lens (35% laser power, 4 × 10 s accumulation time). To account for biological and spectral variations, for each bacterial isolate, three technical replicates were collected from each sample and three biological replicates were acquired from independent samples on different days. Typically, these nine spectra were averaged using GRAMS/Al (version 9.3). For PCA analysis, the raw spectra were normalized to the highest peak of each spectrum and Savitzky–Golay smoothed using Spectragryph optical spectroscopy software (version 1.2.12). Analysis was performed on the spectral region between 300 and 1800 cm^–1^.

### Statistical Analysis

PCA was performed using R version 4.1.2 (R Core Team (2021). R: A language and environment for statistical computing. R Foundation for Statistical Computing, Vienna, Austria (https://www.r-project.org/) and RStudio (version 2021.09.2) Software. Difference (between analytical groups/species) was assessed using distance-based metric (Euclidean distance) and presented as PCA plots displaying variance explained for the first three PCs.

## Results and Discussion

**Figure 1:**
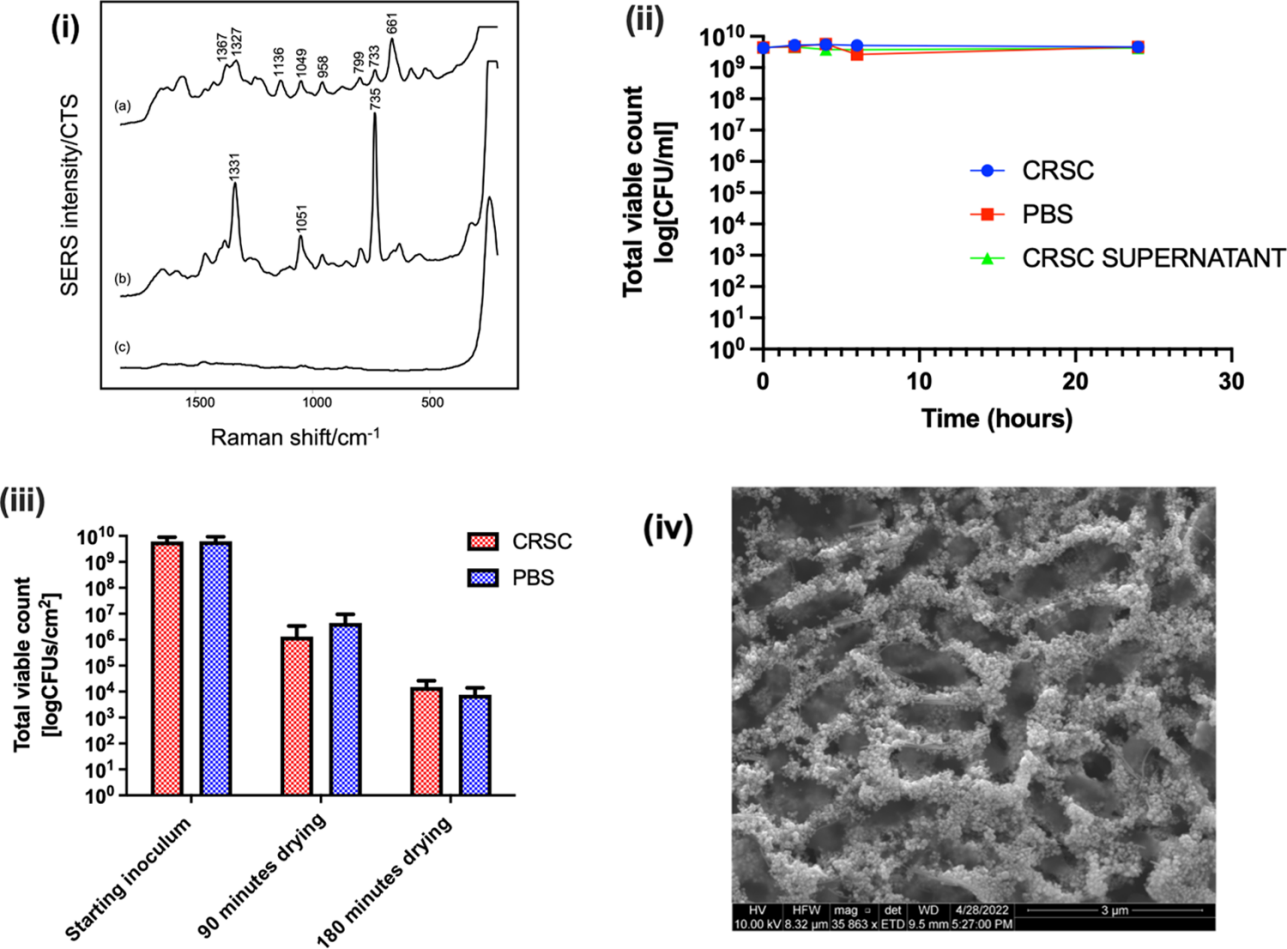
(i) Comparison of the spectra of (a) *P. aeruginosa* AUS 454, with the spectra of the supernatant from the (b) 1st and (c) 3rd wash steps. Spectra are an average of data acquired in triplicate and repeated over 3 days. (ii) Effect of CRSC (●), CRSC supernatant (▲), and PBS (■) on the bacterial density (log_10_CFU/mL) of *P. aeruginosa* AUS 454 over 24 h. (iii) Bacterial density (log_10_CFU/cm^2^) of *P. aeruginosa* AUS 454 after mixing with either CRSC or PBS and dried on an aluminum foil for 90 and 180 min. Dried drops were sonicated for 5 min. Each isolate was replicated in triplicate (error bars represent mean ± SD). (iv) SEM image of *P. aeruginosa* AUS 454 dried with CRSC.

The bacteria used in this study included type strains and clinical isolates from a range of species; these isolates were selected from a larger sample set due to the clinical relevance of the species and because they provide clear examples of the variation observed within the dataset. Figure 1a shows a typical SERS spectrum of a bacterial sample (*Pseudomonas aeruginosa* AUS 454), which highlights that the SERS method used in this study gives spectra with very high S/N ratios even with accumulation times of 40 s. The vibrational bands in the spectra at 799, 735, and 663 cm^–1^ have previously been assigned as nucleotides and their metabolic products. For convenience, we will refer to these bands here as uracil, adenine, and guanine, respectively, while still recognizing that they could arise from one or more different molecular components with similar structures, e.g., adenine-related molecules include adenosine, adenosine monophosphate, adenosine triphosphate, etc. One complicating factor is that the growth media gave very strong SERS signals at the same positions as those found in the spectra of many of the bacteria investigated presumably because both contain chemically similar compounds (Figure S1i). This signal could also be detected in the supernatant produced in the first wash step (Figure 1b) but was dramatically reduced after 3 wash steps (Figures 1c and S1ii).

The data in Figure 1 allow us to address a second question, which has been debated since early studies in this area, and is how do the compounds responsible for the vibrational bands come to be on the surface of the enhancing particles for detection? One possibility is that the spectra are due to compounds that are secreted by bacteria into the surrounding media. However, in the procedure used, the wash steps would remove any cellular metabolites secreted into the growth medium, along with the constituents of the medium itself, which therefore does not contribute to the observed signal (Figure S1ii). This is consistent with the study by Dina et al. who found no contribution in the SERS spectra from compounds used during sample preparation or released by the bacteria into the surrounding media.^12^

Alternatively, under the experimental conditions used, the bacteria may be disrupted so that the intracellular components are released, and the spectra therefore reflect these intracellular components. Indeed, it has been suggested by Cui et al. that silver nanoparticles are toxic to bacteria and cause cell lysis.^20,21^ To determine in our experiments if mixing the bacterial pellet with CRSC caused cell lysis, the bacterial pellet was mixed with CRSC, and the bacterial density was measured over 24 h (Figure 1(ii)). For *P. aeruginosa* AUS 454, there was no decrease in the bacterial density over 24 h in PBS, CRSC supernatant, and CRSC. Further examples from different bacterial species, some of which did show changes, are presented in Figure S2. This was similar to the result found by Kahraman et al. who also noted no difference between the density of *Escherichia coli* and *Bacillus megaterium* mixed with CRSC and that of the control, although in that case, the exposure was only for 30 min.^7^ Nonetheless, silver is well known to be toxic for bacteria; therefore, in this study, the reason for the observed low toxicity may be that the silver concentration was lower than required to cause cell lysis.

In addition, it has been proposed that intracellular components can be released when the bacterial pellet is mixed with CRSC, even in the absence of cell lysis, due to the bacteria being in an unfavorable environment.^7^ In the current study, evidence that mixing the bacteria with CRSC did not cause the release of SERS active molecules (either with or without cell lysis) was provided by an experiment where the bacterial pellet was mixed with CRSC and then centrifuged to give a supernatant, which could be tested using SERS and this was found to give no detectable SERS bands (Figure S4). This is consistent with the results of Kahraman et al. who found some weak bands in the spectra of a similar supernatant, which resembled those of the bacteria but concluded that the intensity was too low for significant contribution to the spectra of bacteria.^7^

In the current SERS experimental protocol, bacterial isolates were mixed with CRSC and pipetted onto the aluminum foil within 10 min for drying. The results above clearly show that it would be unlikely that CRSC would affect viability during the 10 min when the isolates were in solution with the colloid. However, it is possible that lysis could occur during the subsequent drying step, during which the silver concentration increased. Indeed, Kahraman et al. have proposed that intracellular nucleotides were released during the drying of a spotted sample.^7^ To determine if cell lysis occurred during drying, the bacterial pellet was mixed with CRSC or PBS and dried on an aluminum foil for 90 or 180 min, which was the minimum and maximum amount of drying time that was used before SERS measurements were recorded. The samples were then sonicated to remove live bacteria from the aluminum foil. Figure 1(iii) shows the data for 5 min sonication (data recorded for 10 and 20 min sonication was similar, showing that sonication was not causing additional cell death).

Figure 1(iii) shows that when *P. aeruginosa* AUS 454 was mixed with CRSC or PBS and then dried for 90 min, the bacterial density reduced by ∼2 to 3 log units compared to the starting inoculum. It is apparent that it was the drying process that was leading to cell death, rather than contact with the silver nanoparticles since there was little difference in the reduction in density when bacteria were dried in either CRSC or PBS. When the drying time was increased to 180 min, the bacterial density was reduced further but again the levels were similar in the absence or presence of silver nanoparticles. Further examples from different bacterial species are presented in Figure S5. The most probable explanation for this result is that evaporation of the liquid from the droplet increased the salt concentration, which resulted in water moving out of the bacterial cells by osmosis, causing cell death and the release of intracellular components. However, irrespective of the detailed mechanism, the most important observation is that this effect occurred independently of whether nanoparticles were present or not.

The SEM image of *P. aeruginosa* AUS 454, recorded at the perimeter of the dried deposit where the “coffee-ring” forms, is shown in Figure 1(iv). The image shows a matrix composed of silver nanoparticles with voids where the bacteria were located. Further images (Figure S6) showed that the bacteria were present across the whole deposit, but the nanoparticles were mainly concentrated near the edge of the droplet, which created the visible coffee-ring. Closer inspection of the images showed holes in the bacterial isolates, indicating that these cells had lysed. Since SERS spectra were recorded on similar dried samples, it is easy to understand that the signals arise from intracellular compounds, which are released into the surrounding enhancing particles during the drying step.

**Figure 2:**
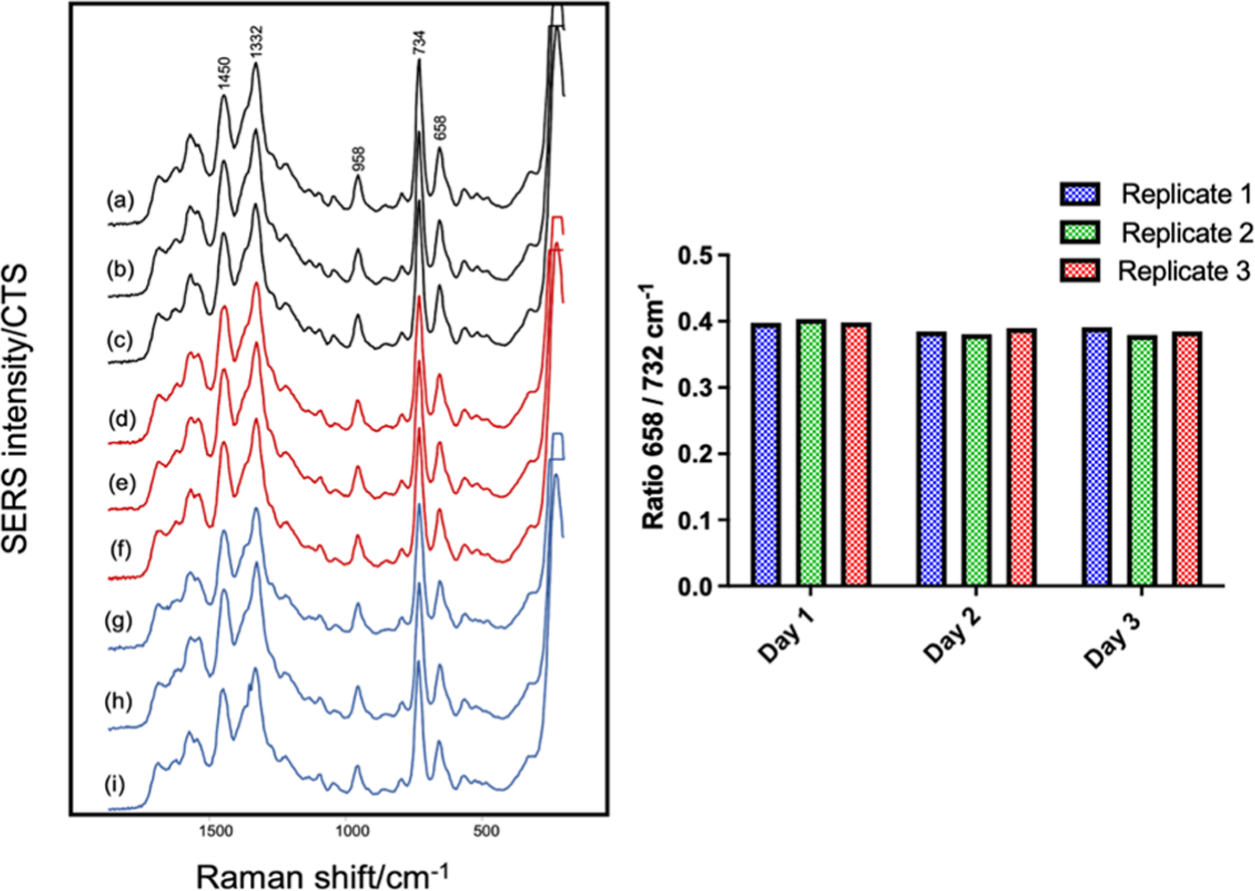
(i) Illustration of the reproducibility of the SERS spectra of *E. coli* UM013 shown as three technical replicates recorded on 3 different days: (a–c) Day 1, (d–f) Day 2, and (g–i) Day 3. (ii) Comparison of the ratio of the relative intensities of the 658:732 cm^–1^ vibrational bands.

Before investigating the effect of microbiological factors on the spectra, it was important to determine the reproducibility of the spectra obtained from the simple mixing of the bacterial pellet and CRSC. For the technical replicates, the SERS measurements were obtained in triplicate from the same sample, and independent technical replicates were then obtained on 3 different days. Exemplar data for *E. coli* UM013 is shown in Figure 2, where the high level of reproducibility within each day and between different days is obvious.

A comparison of the ratio of the relative intensities of two nucleotide bands at 658 and 732 cm^–1^ in the spectra of *E. coli* showed minimal variation within each set of three technical replicates and between replicate data recorded on different days (Figure 2). These spectra highlight that, provided the experimental factors are well-controlled, simple mixing of the bacterial pellet and colloid followed by drying can give very reproducible spectra. Figures S7 and S8 show further examples of the reproducibility of the SERS spectra.

Some previous studies have had success in recording spectra using simple mixing, with some adding the bacteria to a cuvette containing the colloid suspension^22,23^ and others mixing the colloid with the bacterial pellet and drying before taking measurements.^7,24,25^ However, technical replicates were not included in some of these studies and so it was not possible to comment on the reproducibility of the method.^7,22,23^ Nonetheless, Avci et al. and Colnita et al. did include technical replicates, which also showed good reproducibility.^24,25^ In contrast, it has also been stated in the literature that the spectra from samples prepared by simple mixing were inconsistent and not reproducible.^5,9,10^ In the current study, high reproducibility was achieved, which is partly due to the experimental system used. The PerkinElmer instrument has a 100 mW 785 nm external cavity diode laser, which is coupled by a multimode optical fiber into the microscope, resulting in a large 60 μm diameter spot. This allowed the use of a higher laser power than would be possible with diffraction-limited (<1 μm) excitation. More importantly, the larger spot diameter results in sampling over large regions of the sample, which could average out heterogeneity on the <10 μm scale that is apparent in the SEM image shown in Figure 1(iv) and would contribute to the very high reproducibility we have observed.

Establishing that the experimental protocol yields reproducible spectra under fixed experimental conditions meant that the effect of additional microbiological factors could be investigated, as any differences observed in the spectra could be attributed with confidence to differences in the sample. The first variable tested was bacterial density, and, in this study, bacterial isolates were cultured for 6 h and then diluted with Mueller–Hinton broth (MHB) to give samples with different bacterial densities in the range 10^8^–10^9^ CFU/mL.

**Figure 3:**
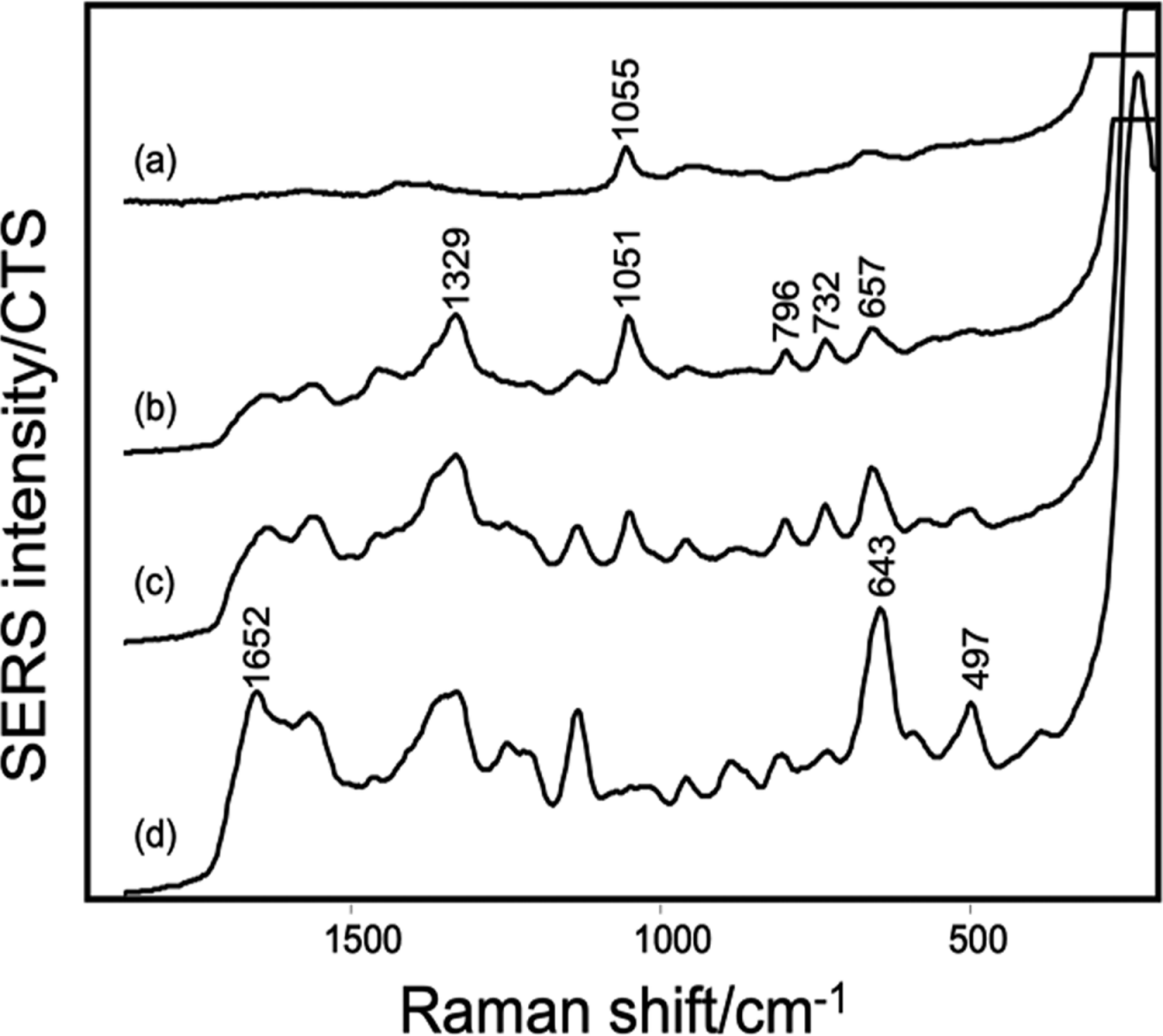
Comparison of the SERS spectra of *P. aeruginosa* AUS 454 at three different bacterial densities ∼(b) 1.8 × 10^8^, (c) 3.2 × 10^8^, and (d) 1.6 × 10^9^ CFU/mL. Spectra shown are averages of data acquired in triplicate and repeated over 3 days. Blank CRSC control (a) was included for comparison. Spectra shown are offset but with the same intensity scale.

Figure 3 shows two spectra of *P. aeruginosa* AUS 454 with bacterial densities of ∼10^8^ CFU/mL, which were similar to each other but very different from the spectra obtained at ∼10^9^ CFU/mL. This sample showed a particularly large difference, but it emphasizes that the bacterial density does need to be controlled if spectra are to be compared. A further example of *Staphylococcus aureus* is presented in Figure S9. The origin of the differences in the spectra is presumably associated with the interplay between the total concentration and the relative binding affinities of the different compounds (believed to be predominantly nucleotides and purine derivatives). At high concentrations, competition has a crucial role in determining which molecules bind to the surface of the SERS substrates. The molecules with the highest binding affinities will outcompete other molecules, occupying a higher proportion of the available surface sites and therefore giving the strongest SERS signal.^26,27^ Conversely, at low bacterial densities (i.e., only sufficient to give near monolayer or even sub-monolayer coverage), all of the available molecules can bind to the SERS substrate, without competition, so that the signal is not distorted by the effects of different binding affinities. In a previous study, differences were observed in the spectra of *E. coli* at different bacterial densities, but the reason was not determined.^10^ More generally, in studies where the bacterial density has been considered, the samples were typically adjusted to have a consistent number of CFU/mL.^19,28,29^ Moreover, in many studies where bacterial colonies were collected with a sterile loop, no adjustment was made for bacterial density.^7,16,24,30^

The next microbiological factor investigated was the effect of growth phases on the spectra of bacterial isolates. Some previous studies have reported differences in the spectra from samples cultured for different times,^7,16,24^ but since these studies did not adjust the samples to the same bacterial density at each time point, it is possible that the observed differences in the spectra could be due to differences in bacterial density rather than the phase of growth. In the current study, bacterial isolates were cultured for 3, 6, and 24 h (i.e., early exponential, late exponential, and stationary phase) and adjusted to the same approximate CFU/mL before analysis. Again, several different bacterial species were investigated and differences in the effect (or lack of effect) of the growth phase were found.

**Figure 4:**
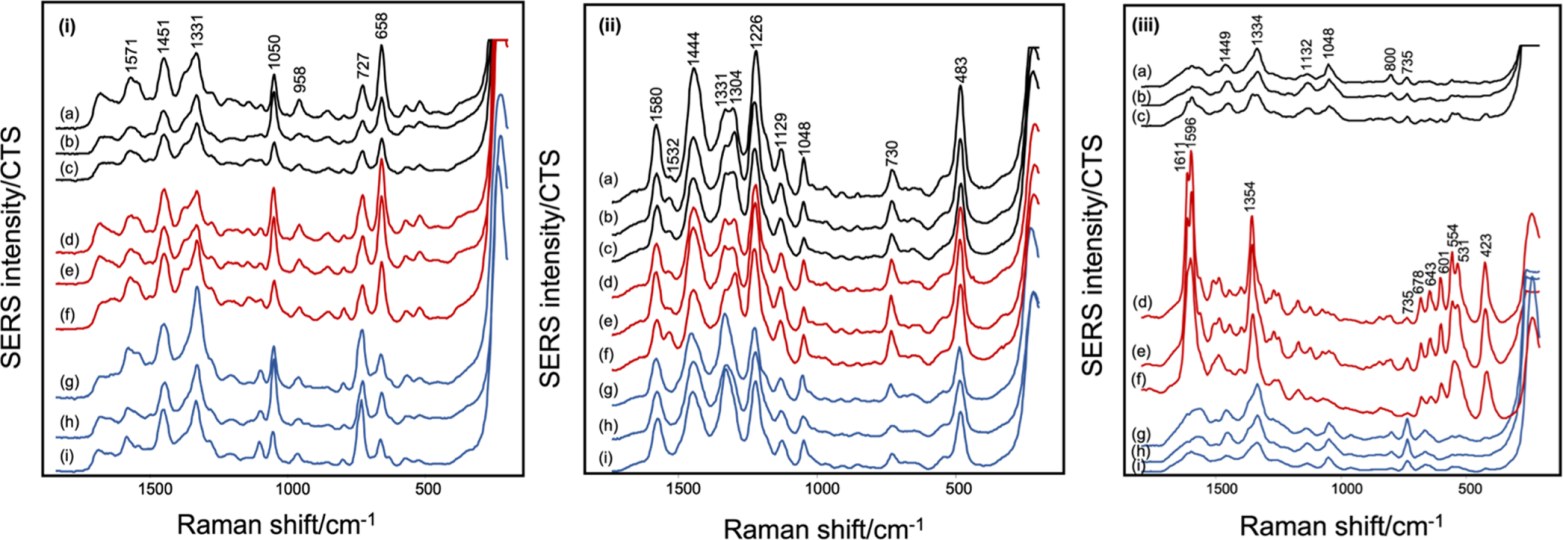
Data showing the effect of the growth phase on the spectra of (i) *A. xylosoxidans* B064 V2S2F, (ii) *S. pneumoniae* CF 108 T6, and (iii) *P. aeruginosa* PAO1 at (a–c) 3 h, (d–f) 6 h, and (g–i) 24 h on Day 1, 2, and 3, respectively. All spectra are the average of three technical replicates acquired from the same independent sample. Spectra shown are offset but with the same intensity scale.

Figure 4(i) shows spectra for *Achromobacter xylosoxidans*, where the relative intensities of the 727 and 658 cm^–1^ nucleotide bands at 3 and 6 h were similar but were very different at 24 h. This change in relative intensities of nucleotide bands was not unexpected since the differences in the band ratios presumably reflect differences in the metabolic processes. Another example showing the effect of the phase of growth on the spectra of *Stenotrophomonas maltophilia* is given in Figure S10, which again shows similar SERS spectra at 3 and 6 h but very different spectra at 24 h. This data is consistent with the fact that bacterial isolates are dividing and metabolically active during the first 6 h of growth, producing DNA, RNA, and cell wall components.^31^ However, at 24 h, the metabolic activity of the bacterial isolates had likely slowed, which may have resulted in the observed differences in the spectra.^31^

The consistency of the 3 and 6 h spectra might suggest that for all bacteria there are broad time ranges within which consistent spectra can be obtained but that exponential and stationary phases are distinct. However, it is important not to extrapolate general conclusions from limited studies. For example, as shown in Figure 4(ii), the spectra of *Streptococcus pneumoniae* CF 108 T6 were very similar at all three time points, with only small differences in the relative intensity of the bands at ca. 1300–1330 cm^–1^, which demonstrates that changing the growth phase may not lead to detectable spectral differences for all bacterial species. Conversely, the spectra recorded after 6 h for *P. aeruginosa* PA01, Figure 4(iii), were dominated by the very distinct characteristic pyocyanin (PCN) bands but those at 3 and 24 h have no significant PCN features. This supports the assertion that the experimental method used detects intracellular compounds, rather than secreted extracellular metabolites; at 24 h, the culture medium is a green/blue color due to PCN, but this is removed during the washing steps so that no PCN is detected. As the same procedure was used for the spectra recorded at 6 h, PCN secreted into the media would have been removed; therefore, the PCN detected must have been intracellular. The spectra for *P. aeruginosa* PA01 (Figure 4(iii)) were recorded on three separate occasions to demonstrate that the appearance of PCN at 6 h was a reproducible effect, rather than a random change in the way the culture grew on a particular day. More generally, recording biological replicates is important because bacteria are living organisms and do not necessarily respond or grow the same way on different days, even if the growth conditions are kept the same. Many studies have only reported technical replicates and recorded numerous spectra from the same sample.^10,14,32−34^ Some have performed biological replicates on one bacterial isolate as a proof of concept and then only performed technical replicates on remaining isolates,^24,28,29^ while others have performed biological replicates and variation was observed in the spectra.^35,36^

**Figure 5:**
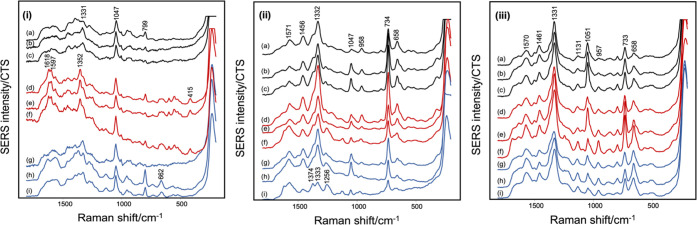
Data showing the effect of biological replicates on the spectra of (i) *P. aeruginosa* AUS 253, (ii) *H. influenzae* B077 V2S2A, and (iii) *B. multivorans* B007 V1S1B at (a–c) 3 h, (d–f) 6 h, and (g–i) 24 h on Day 1, 2, and 3, respectively. All spectra are the average of three technical replicates acquired from the same independent sample. Spectra shown are offset but with the same intensity scale.

In the context of the current study, it is useful to understand the link between the experimental conditions (specifically culture time) and biological variation; therefore, the spectra of three biological replicates at different culture times were recorded for a broad range of different bacterial species, to determine the extent of biological variation shown by different bacterial species. The SERS spectra shown in Figure 4, together with the five additional bacterial isolates shown in Figures 5, S10, and S11, illustrate that some bacteria show little biological variation for any given culture time, e.g., *A. xylosoxidans* B064 V2S2F, *P. aeruginosa* PA01 (Figure 4(i, iii)), but others, e.g., *P. aeruginosa* AUS 253, *Haemophilus influenzae* B077 V2S2A, *S. maltophilia* B035 V4S2J, and *P. aeruginosa* AUS 454 (Figures 5(i, ii), S10 and S11), show differences. In this dataset, it was typically the spectra at 24 h that showed the largest biological variation. There were exceptions, such as *Burkholderia multivorans* B007 V1S1B (Figure 5(iii)), which showed variation at 6 h.

For the majority of the investigated samples from our dataset, the 6 h time point produced the most consistent spectra and PCN produced from *P. aeruginosa* isolates could be detected, so this was chosen as the standard culture time for future experiments. However, it is important to highlight that the time point and bacterial density that we have chosen to use as a standard may not be optimum for every bacterial species/isolate because the aim was to try to find the conditions that gave the most reproducible spectra.

**Figure 6:**
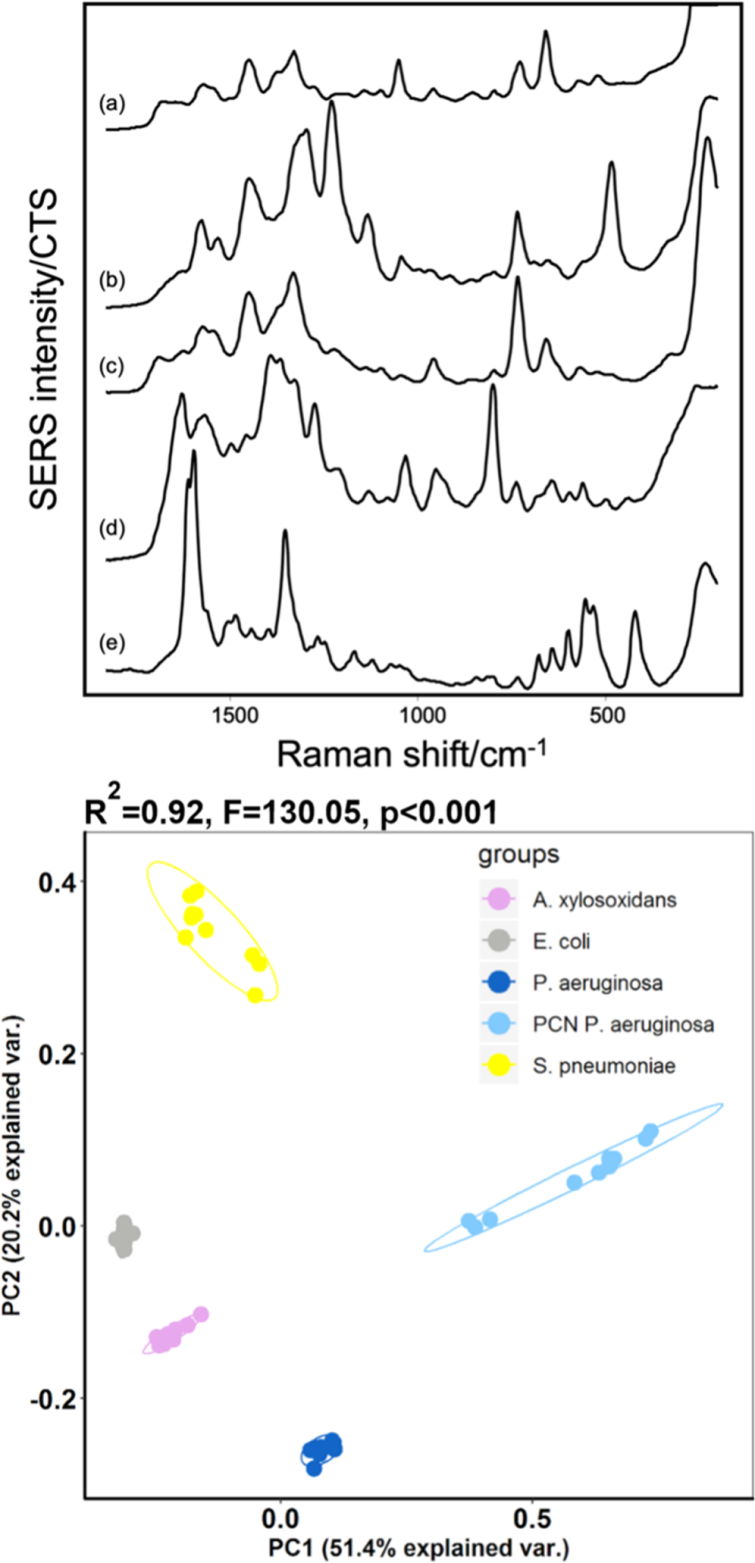
SERS spectra of five different bacterial isolates. (a) *A. xylosoxidans* B064 V2S2F, (b) *S. pneumoniae* CF 108 T6, (c) *E. coli* UM013, (d) *P. aeruginosa* B004 V4E2E, and (e) PCN producing *P. aeruginosa* PA01. Data are an average of nine spectra, and three technical replicates obtained on 3 different days. PCA plot includes the SERS spectra of the technical and biological replicates from the five isolates.

Several previous studies have used multivariate data analysis to discriminate between the spectra of different bacterial species.^12,14,24,25,35,37^ However, while many of these studies were successful, this was typically in the context of very limited experiments where the number of isolates and/or species was small and there were limited technical and/or biological replicates. This makes discrimination between samples easier to demonstrate; however, it does raise the question of how successful such methods would be with larger and more complex sets of samples where there is a higher probability of similarity between spectra from different species. Having established that high degrees of spectral reproducibility can be achieved, we determined the extent to which residual variation, which cannot be eliminated by standardizing culture time and bacterial density, impacts the ability to discriminate between different bacterial species. In this case, for illustration, five bacterial isolates whose average spectra (three technical replicates on 3 separate days) are quite distinct from each other were chosen. The technical and biological replicates for each isolate were included in the PCA analysis, whose results are shown in Figure 6.

**Figure 7:**
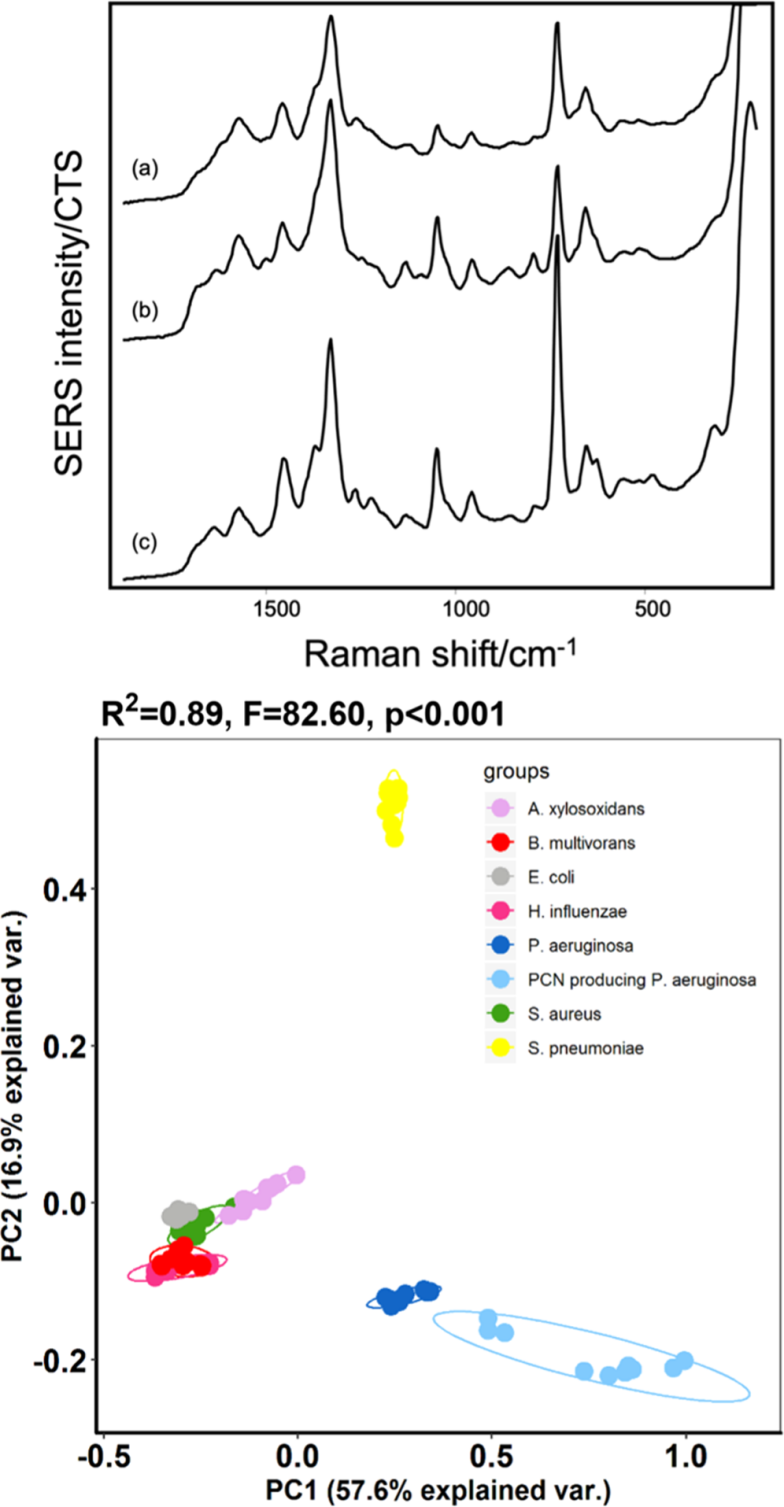
SERS data for three bacterial isolates with very similar spectra. (a) *H. influenzae* B077 V2S2A, (b) *B. multivorans* B007 V1S1B, and (c) *S. aureus* 15A. Data are an average of nine spectra, and three technical replicates obtained on 3 different days. PCA plot includes the SERS spectra of the technical and biological replicates from the eight isolates.

The PCA plot, Figure 6, shows that the clusters were very tight for some isolates, as the spectra were highly reproducible, and these isolates also showed low biological variation. However, the spectra of both *S. pneumoniae* and *P. aeruginosa* showed biological variation that dramatically increased the size of the clusters. As the separation between these two species was large, there was still a clear separation between all of the bacterial species using two PCs, even when biological replicates were included. These results are consistent with some other studies in the literature, where good discrimination between several species was easily obtained with two PCs. However, if additional bacterial species need to be included in the analysis, it may make discrimination more challenging. Here, for the purposes of illustration, three further bacterial species were included in the PCA analysis. Their spectra were similar to each other and also similar to *E. coli* (Figure 6). When all eight bacterial isolates were included, the overlap was observed between *B. multivorans*, *H. influenzae*, *E. coli,* and *S. aureus*, as shown in Figure 7.

Figure 7 is an extended PCA plot, with two PCs, which includes data from the larger set of samples and shows overlap between several of the species. While further visualization of the dataset with the first three PCs (Figures S13 and S14) showed more separation of the clusters for *A. xylosoxidans*, *B. multivorans*, *H. influenzae*, *S. aureus,* and *E. coli*, it was still not sufficient to discriminate between all of the species. Overall, this small study demonstrates that the degree to which SERS can be used to discriminate between bacterial species depends heavily on the nature of the sample set chosen and which experimental variables are systematically controlled. Clearly, it is straightforward to discriminate between samples that have very different spectra if technical replicates are very reproducible since the clusters do not overlap even when biological replicates are included. However, when the samples give very similar spectra, even good technical reproducibility is not sufficient to allow ready separation since biological variation will always be present and must be included in the testing procedure. Of course, if the experimental conditions are relaxed so that effects of the bacterial density or growth phase are also not controlled, this will exacerbate the problem further. Furthermore, as the data shown above, different isolates from the same species can be very markedly different from each other. These considerations mean that a very large dataset comprising many different bacterial isolates from a wide range of species will need to be studied before it is possible to determine if SERS can be used as a general method to discriminate between bacterial species and/or strains.

## Conclusions

This study has demonstrated that experimental and microbiological variations were independent factors and highlighted the importance of considering both factors separately when analyzing bacteria using SERS. While it was not possible to comment on the day-to-day reproducibility of other methods published in the literature, this study has demonstrated excellent reproducibility of the spectra with simple mixing of the bacterial pellet and CRSC, providing experimental factors were well-controlled. The development of a reproducible SERS method meant that microbiological factors could be investigated and differences observed in the spectra could be attributed with confidence to differences between the samples. The clear observations that spectra change with the bacterial density and phase of growth suggest that direct identification of pathogenic bacteria in clinical samples may be difficult, as these parameters would not be well-controlled. Similarly, the observation that some isolates give noticeably different spectra even when cultured under nominally identical conditions may have implications for identification from clinical samples, as growth conditions are likely to vary significantly between samples. Finally, while some bacterial species gave distinct spectra, there were multiple examples where the differences between different species were much smaller than the differences that were associated with the growth phase or bacterial density. Overall, this study illustrates that it is important to avoid drawing general conclusions about bacterial SERS based on studies using small numbers of samples since different bacterial species/isolates act very differently and conclusions that are entirely valid for a given sample set may not be correct when different sets of bacteria are studied.

## Supporting Information Available

Supporting methods; table showing media and incubation conditions for bacteria; figures showing spectra of culture media, CRSC and supernatant after each wash step (Figure S1), effect of CRSC on the bacterial density (Figure S2), images of recultured bacteria after inoculation with CRSC (Figure S3), spectra of the supernatant of the bacteria/colloid mixture (Figure S4), effect of drying in PBS or CRSC on the bacterial density (Figure S5), SEM images of bacteria (Figure S6), spectra of technical replicates (Figure S7 and 8), spectra of *S. aureus* at different bacterial densities (Figure S9), spectra showing the effect of the growth phase (Figure S10 and S11), PERMANOVA analysis (Figure S12), and PCA plot with two PCs and three PCs (Figure S13 and S14) (PDF)
