# Supplementary material for: Characterization of Bacteria Using Surface-Enhanced Raman Spectroscopy (SERS): Influence of Microbiological Factors on the SERS Spectra
Source: Anal Chem. 2022 Jun 17;94(26):9327–35. doi: 10.1021/acs.analchem.2c00817 (PMC9260712; doi:10.1021/acs.analchem.2c00817)
Supplement: Supplementary file 1 — ac2c00817_si_001.pdf [file ac2c00817_si_001.pdf]

Supporting Information

Characterization of bacteria using surface-enhanced Raman Spectroscopy (SERS): influence of microbiological factors on the SERS spectra

Danielle M. Allen [a*], Gisli G. Einarsson [b], Michael M. Tunney [a], Steven E. J. Bell [c*]

[a] School of Pharmacy, Queen's University Belfast, 97 Lisburn Road, Belfast, Northern Ireland, BT9 7BL

[b] Centre for Experimental Medicine, School of Medicine, Dentistry and Biomedical Sciences, Queen's University Belfast, 97 Lisburn Road, Belfast, Northern Ireland, BT9 7BL

[c] School of Chemistry and Chemical Engineering, Queen's University Belfast, University Road, Belfast, Northern Ireland, BT7 1NN.

*Corresponding author e-mail: danielle.allen@qub.ac.uk and s.bell@qub.ac.uk

Table of Contents

Supplementary Method

Pour plate assay……………………………………………………………………………S3

Multivariate-permutational analysis (PERMANOVA) ……………………………………...S3

Supplemental Tables

Table S1. Media and incubation conditions used for bacterial isolates………………………S4

Supplemental Figures

Figure S1. (i) SERS spectra of culture media and CRSC. **(ii)** Comparison of the spectra of *P. aeruginosa* AUS 454 with the spectra of the supernatant after each wash step……………….S5

Figure S2. Shows the effect of CRSC, CRSC supernatant and PBS on the bacterial density (log_{10}CFU/mL) of 8 bacterial isolates over 24h…………………………...……………….S6

Figure S3. Images of re-cultured bacteria and their control after 24 h………..……………….S7

Figure S4. Comparison of the spectra of *P. aeruginosa* AUS 454, with the spectra of the supernatant of the bacteria/colloid mixture…...…………………………...……………….S8

Figure S5. Bacterial density (log_{10}CFU/cm^2) of 8 bacterial isolates after mixing with either CRSC or PBS and dried on aluminium foil for 90 and 180 mins..……………………………S9

Figure S6. SEM images of 3 bacterial species dried with CRSC…..………….………………S10

Figure S7 and S8. Illustration of the reproducibility of the technical replicates of 8 bacterial isolates and comparison of the ratio of the relative intensities of 2 vibrational bands......S13-14

Figure S9. Comparison of the SERS spectra of *S. aureus* at different bacterial densities..….S15

Figure S10. Data showing the effect growth phase on SERS spectra of *S. maltophilia* B035 V4S2J……………………………...…………………………...………………..…S16

Figure S11. Data showing the effect growth phase on SERS spectra of *P. aeruginosa* AUS 454……………………..…………………………………..…………………………S17

Figure S12. PCA plots annotated with the results from the PERMANOVA analysis………S18

Figure S13. PCA plot illustrating the relationship between the SERS spectra of 8 bacterial isolates with 2 principal components (PC) (i) PC1 *v* PC3 and (ii) PC2 v PC3……...……….S19

Figure S14. PCA plot illustrating the relationship between the SERS spectra of 5 bacterial species with three principal components. ………………………………...…………….…S20

Supplemental method

Pour Plate Assay

To confirm that the CRSC was not toxic to the bacterial isolates, pour plates were prepared. The molten agar was mixed with 1 mL of OD 0.3 bacterial inoculum, 80 µL of CRSC was streaked onto the agar surface and incubated at 37 °C for 24 hrs.

Multivariate-permutational analysis (PERMANOVA)

Differences between species groups were evaluated by multivariate-permutational analysis (PERMANOVA) as implemented within the ADONIS function from the vegan-package (version 2.5-7) in R with 999 permutations.

Table S1. Media and incubation conditions used for bacterial isolates

**Bacterial species**	**Isolate code**	**Source**	**Solid Media**	**Liquid Media**	**Temp (°C)**	**Incubation atmosphere**	**Speed (rpm)**
P. aeruginosa	PAO1	Reference strain	MHA	MHB	35-37	Air	180
	AUS 255	Endotracheal aspirate sample					
	AUS 454	Bronchiectasis sputum					
	B004 V4E2E	CF sputum					
S. aureus	15A	Bronchoalveolar lavage	MHA	MHB	35-37	Air	180
A. xylosoxidans	B064 V2S2F	CF sputum	Blood agar	MHB	35-37	Air	180
E. coli	UM013	Urinary tract infection	MHA	MHB	35-37	Air	180
S. maltophilia	B035 V4S2J	CF sputum	Blood agar	MHB	35-37	Air	180
S. pneumoniae	CF 108 T6	CF sputum	Blood agar	BHI	35-37	5% $C0_2$	0
B. multivorans	B007 V1S1B	CF sputum	Blood agar	MHB	35-37	Air	180
H. influenzae	B077 V2S2A	CF sputum	BCA	BHI + HTM supplement	35-37	5% $C0_2$	0

MHA (Mueller-Hinton agar), MHB (Mueller-Hinton broth), Blood Chocolate Agar (BCA), BHI (Brain Heart Infusion broth) and Haemophilus Test Medium (HTM)

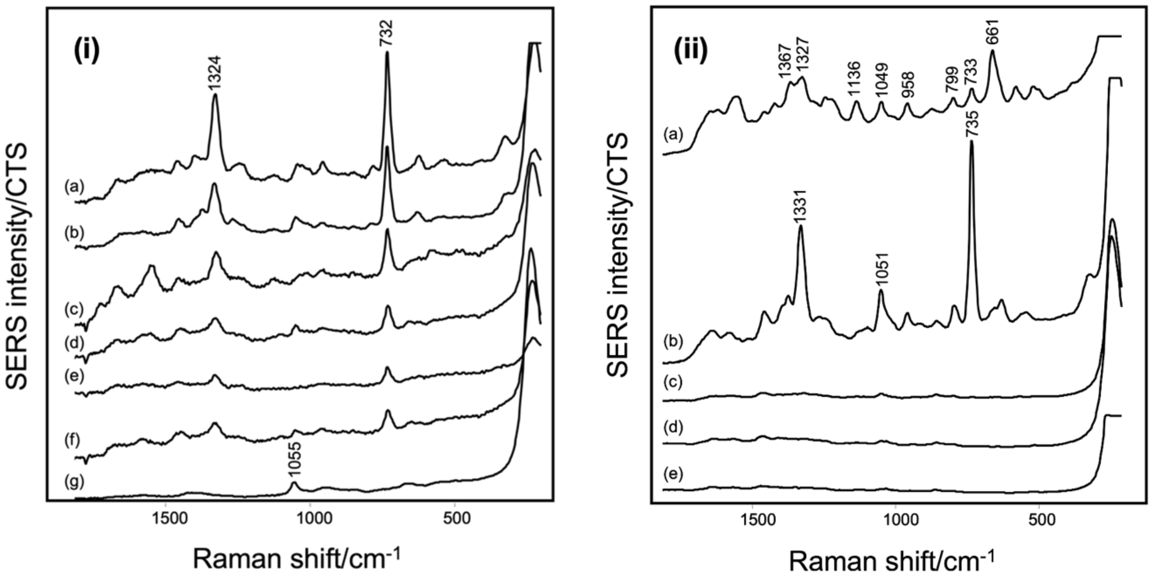

Figure S1. (i) SERS spectra of (a) MHB diluted 10x (b) MHB diluted 100x (c) BHI diluted 10x (d) BHI diluted 100x (e) BHI supplemented with HTM diluted 10x (f) BHI supplemented with HTM diluted 100x and (g) CRSC. **(ii)** Comparison of the spectra of (a) *P. aeruginosa* AUS 454, with the spectra of the supernatant after each wash step: (b) 1st (c) 2nd (d) 3rd. Blank CRSC control (e) was included for comparison. Spectra shown are averages of data acquired in triplicate and repeated on 3 days.

The band at 1050 cm^{-1} in the spectra is due to residual nitrate which is present in the colloid since it was prepared using $AgNO_3$ as the source of the Ag^+ ions.

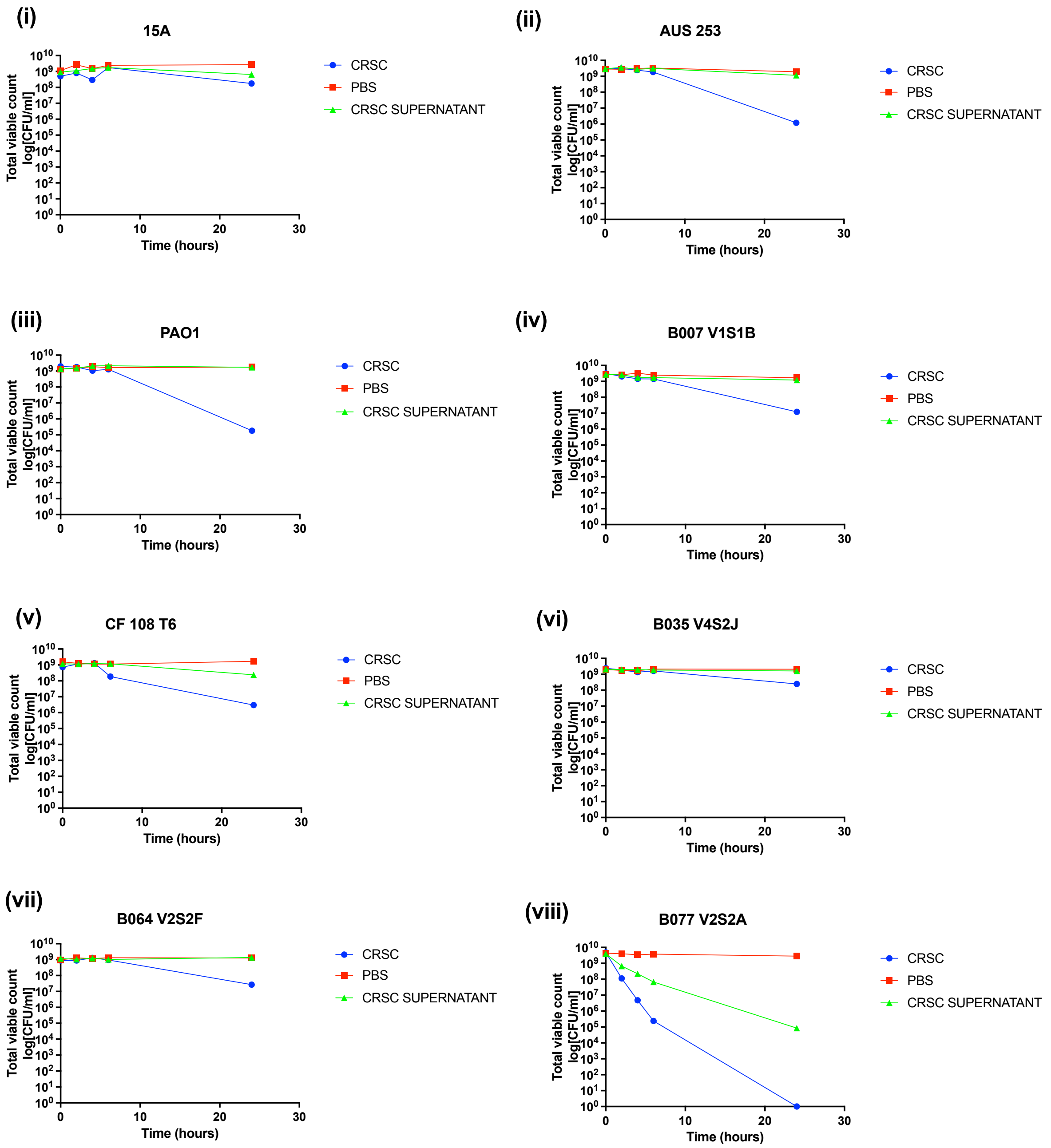

Figure S2. Shows the effect of CRSC (●), CRSC supernatant (▲) and PBS (■) on the bacterial density (log_{10}CFU/mL) of (i) *S. aureus* 15A (ii) *P. aeruginosa* AUS 253 (iii) *P. aeruginosa* PA01 (iv) *B. multivorans* B007 V1S1B (v) *S. pneumoniae* CF108T6 (vi) *S. maltophilia* B035 V2S2J (vii) *A. xylosoxidans* B064 V2S2F and (viii) *H. influenzae* B077 V2S2A over 24h.

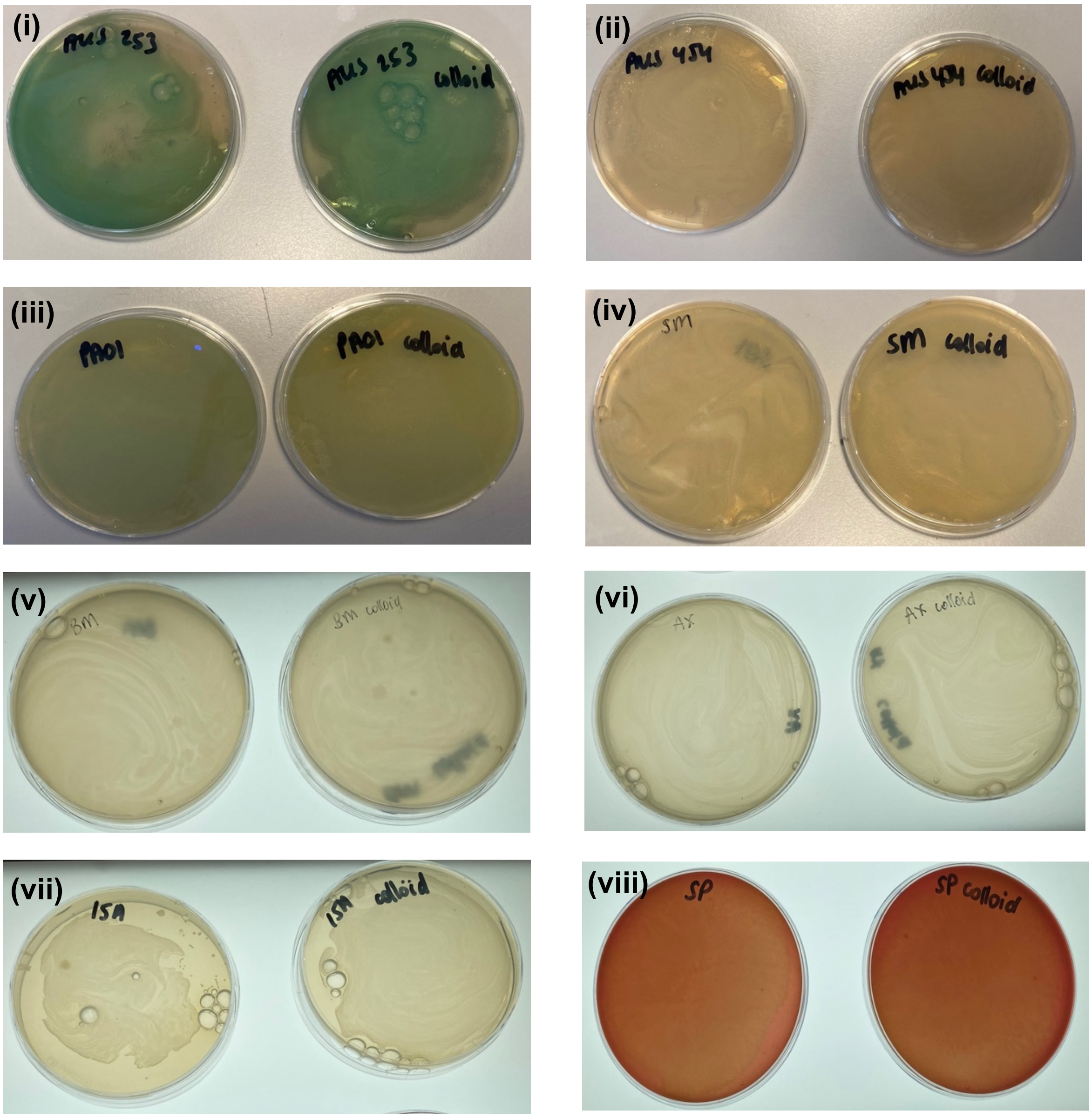

Figure S3. Images showing the growth of bacterial isolates after 24 hours (i) *P. aeruginosa* AUS 253, (ii) *P. aeruginosa* AUS 454, (iii) *P. aeruginosa* PA01, (iv) *S. maltophilia* B035 V2S2J, (v) *B. multivorans* B007 V1S1B, (vi) *A. xylosoxidans* B064 V2S2F, (vii) *S. aureus* 15A and (viii) *S. pneumoniae* CF108T6. The left-hand agar plate shows the control (inoculated with bacteria only and the right-hand agar plate shows bacteria inoculated with CRSC.

The images in Figure S3 showed growth of the bacterial isolates after 24 hours on agar plates after inoculation with CRSC, this further confirmed the results of the viability experiments in Figure 1(ii) and Figure S2.

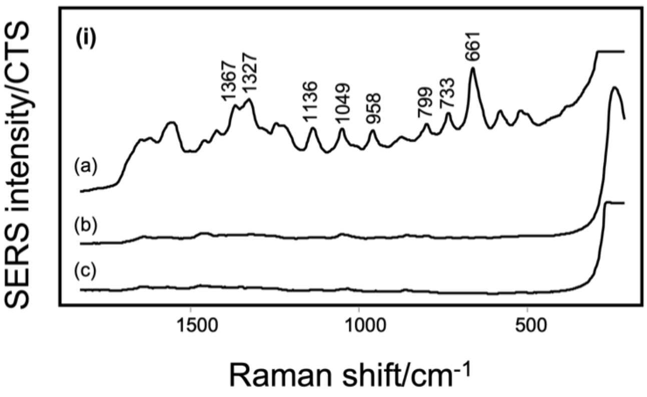

Figure S4. Comparison of the spectra of (a) *P. aeruginosa* AUS 454, with the spectra of the supernatant of the bacteria/colloid mixture (b). Blank CRSC control (c) was included for comparison.

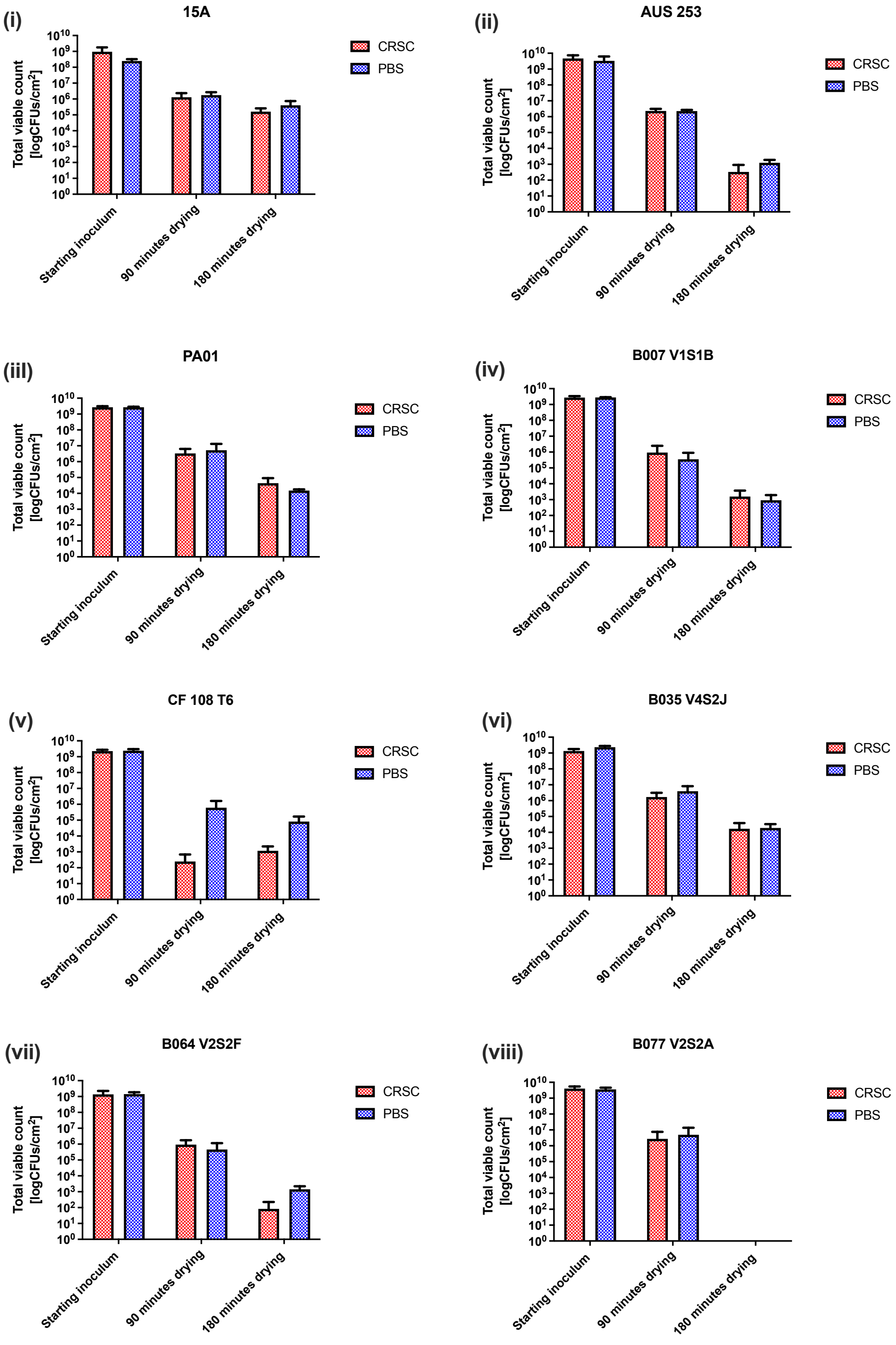

Figure S5. Bar graph showing the bacterial density ($log_{10}CFU/cm^2$) of (i) *S. aureus* 15A (ii) *P. aeruginosa* AUS 253 (iii) *P. aeruginosa* PA01 (iv) *B. multivorans* B007 V1S1B (v) *S. pneumoniae* CF108T6 (vi) *S. maltophilia* B035 V2S2J (vii) *A. xylosoxidans* B064 V2S2F and (viii) *H. influenzae* B077 V2S2A after mixing with either CRSC or PBS and dried on aluminium foil for 90 and 180 mins. Dried drops were sonicated for 5 mins. The starting inoculum of each individual bacterial isolate in CRSC and PBS was calculated. Each isolate was replicated in triplicate (error bars represent mean ± SD).

a.

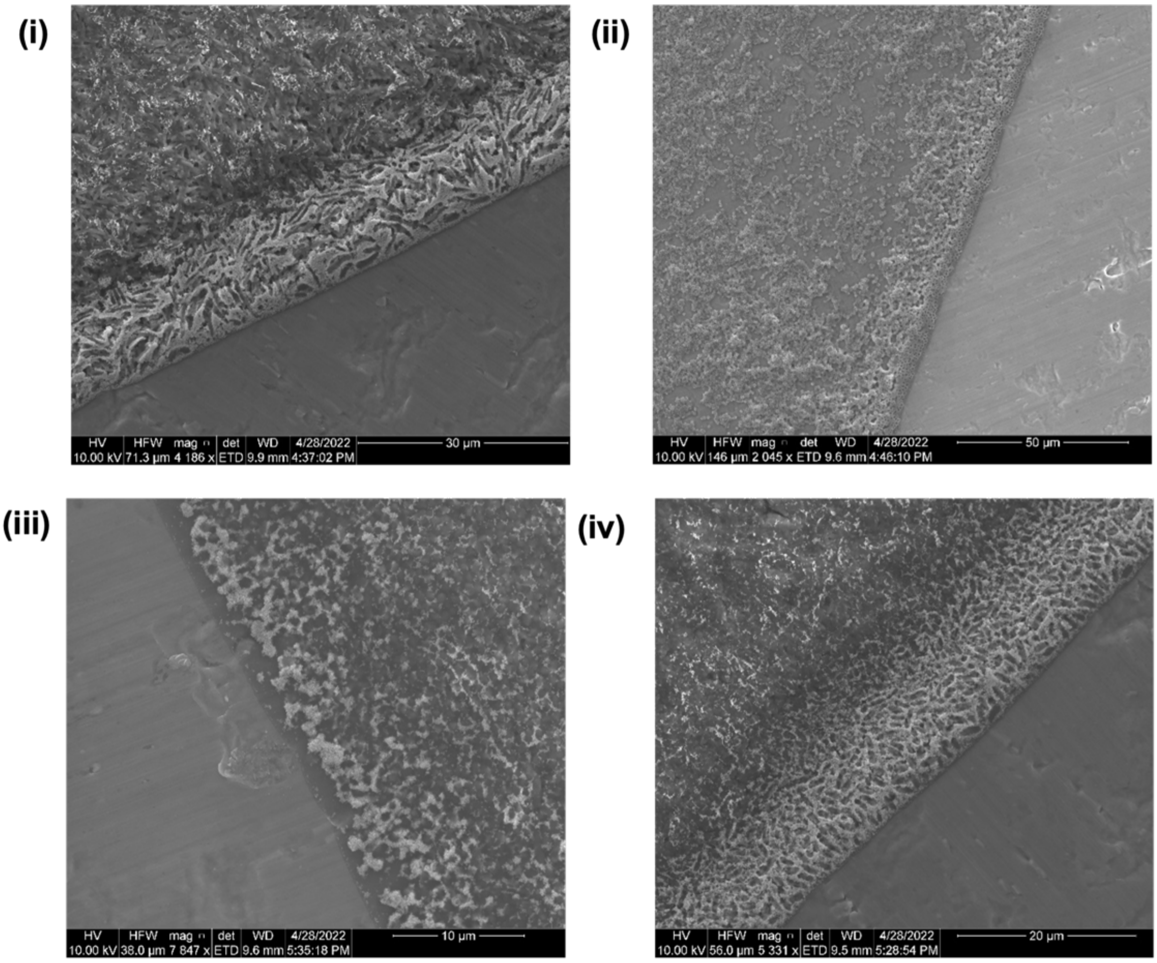
(i)
HV HFW mag det WD 4/28/2022
10.00 kV 71.3 µm 4 186 x ETD 9.9 mm 4:37:02 PM
30 µm
(ii)
HV HFW mag det WD 4/28/2022
10.00 kV 146 µm 2 045 x ETD 9.6 mm 4:46:10 PM
50 µm
(iii)
HV HFW mag det WD 4/28/2022
10.00 kV 38.0 µm 7 847 x ETD 9.6 mm 5:35:18 PM
10 µm
(iv)
HV HFW mag det WD 4/28/2022
10.00 kV 56.0 µm 5 331 x ETD 9.5 mm 5:28:54 PM
20 µm

b.

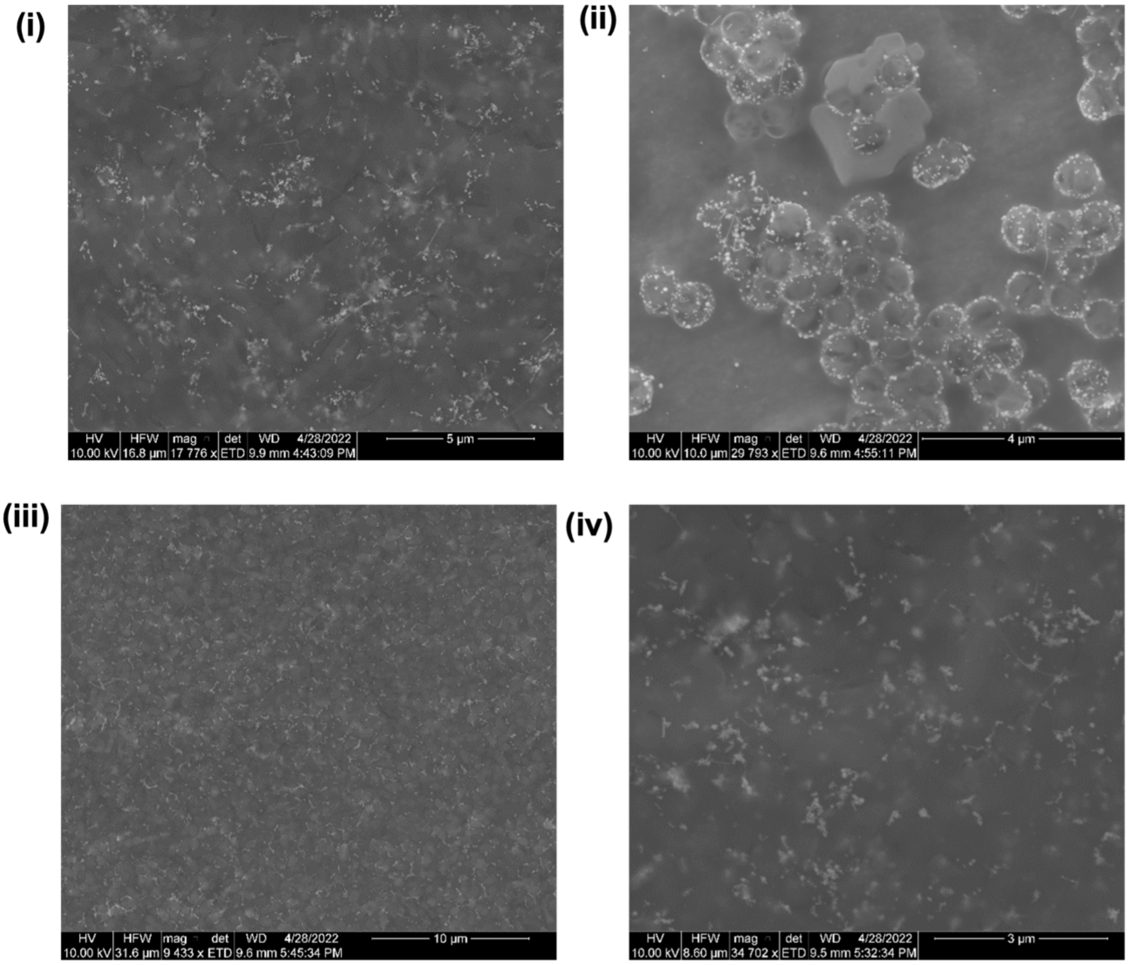
(i)
HV 10.00 kV HFW 16.8 µm mag 17 776 x det ETD WD 9.9 mm 4/28/2022 4:43:09 PM
5 µm
(ii)
HV 10.00 kV HFW 10.0 µm mag 29 793 x det ETD WD 9.6 mm 4/28/2022 4:55:11 PM
4 µm
(iii)
HV 10.00 kV HFW 31.6 µm mag 9 433 x det ETD WD 9.6 mm 4/28/2022 5:45:34 PM
10 µm
(iv)
HV 10.00 kV HFW 8.60 µm mag 34 702 x det ETD WD 9.5 mm 4/28/2022 5:32:34 PM
3 µm

c.

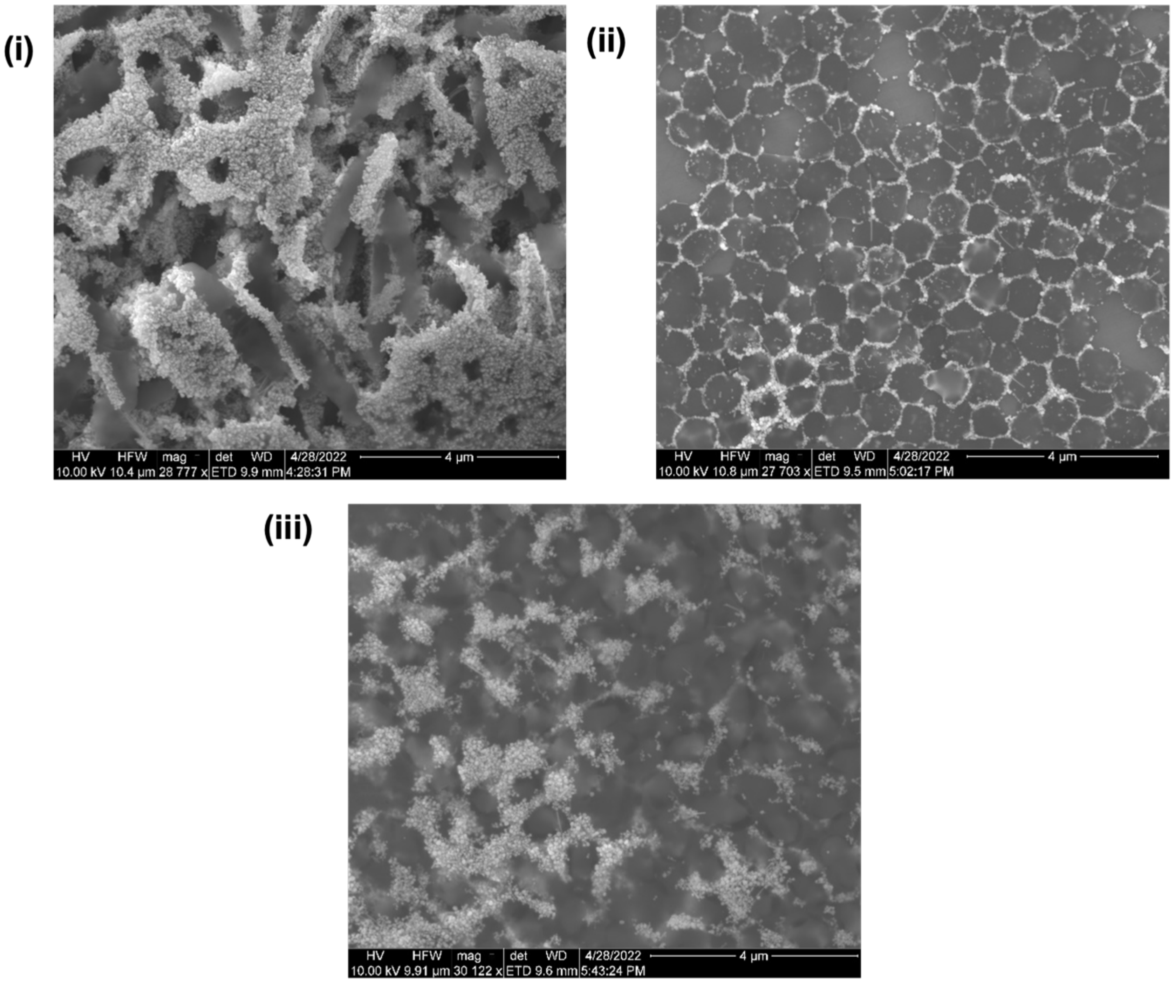

Figure S6. SEM images of (i) *A. xylosoxidans* B064 V2S2F, (ii) *S. aureus* 15A, (iii) *S. pneumoniae* CF 108 T6 and (iv) *P. aeruginosa* AUS 454 dried with CRSC captured at a) the edge of the droplet, b) the center of the droplet, and c) enhanced image at the edge of the droplet.

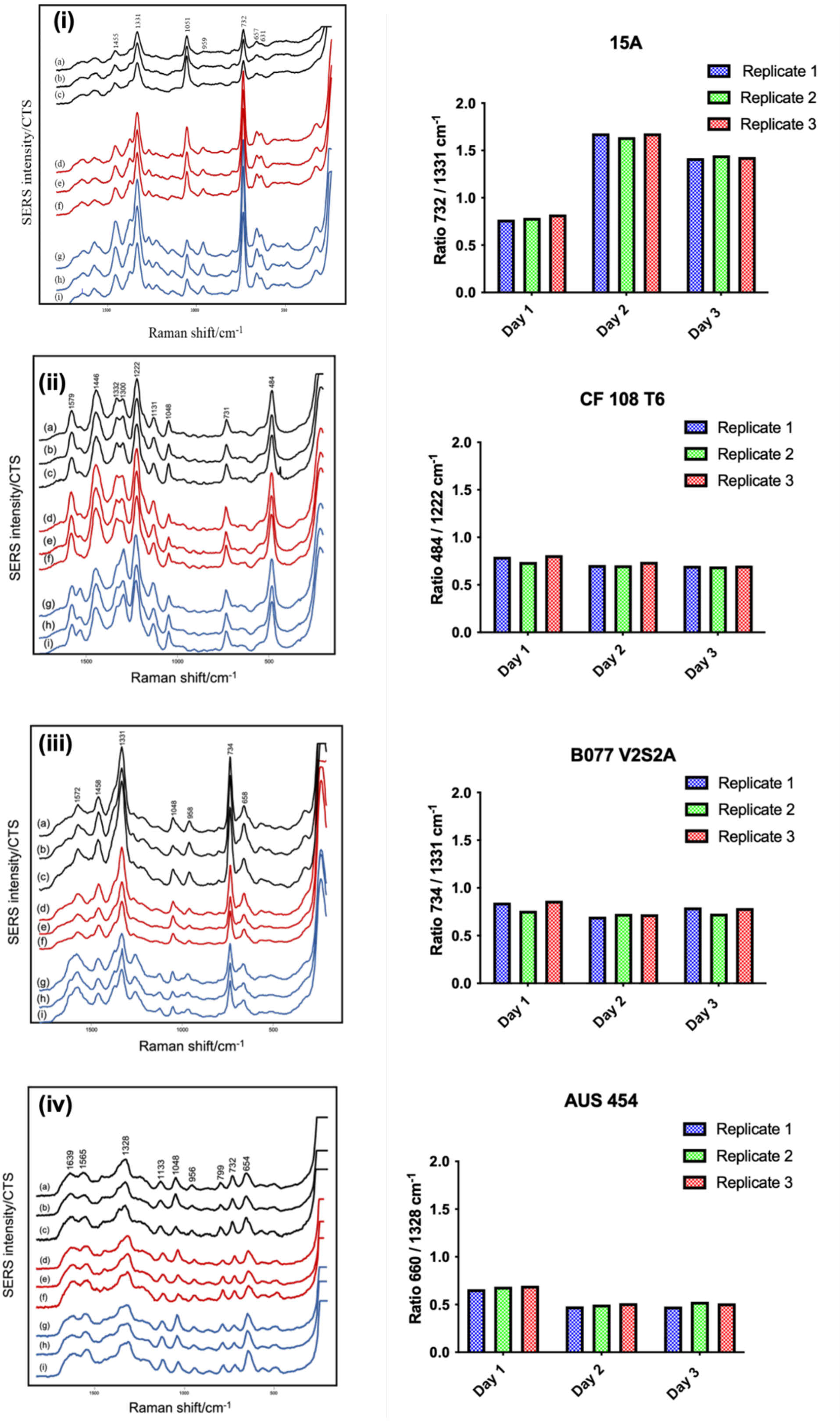

Figure S7. Illustration of the reproducibility of the SERS spectra of (i) *S. aureus* 15A, (ii) *S. pneumoniae* CF108T6, (iii) *H. influenzae* B077 V2S2A, (iv) *P. aeruginosa* AUS 454, shown as 3 technical replicates recorded on 3 different days: (a-c) Day 1, (d-f) Day 2 and (g-i) Day 3 and comparison of the ratio of the relative intensities of two vibrational bands.

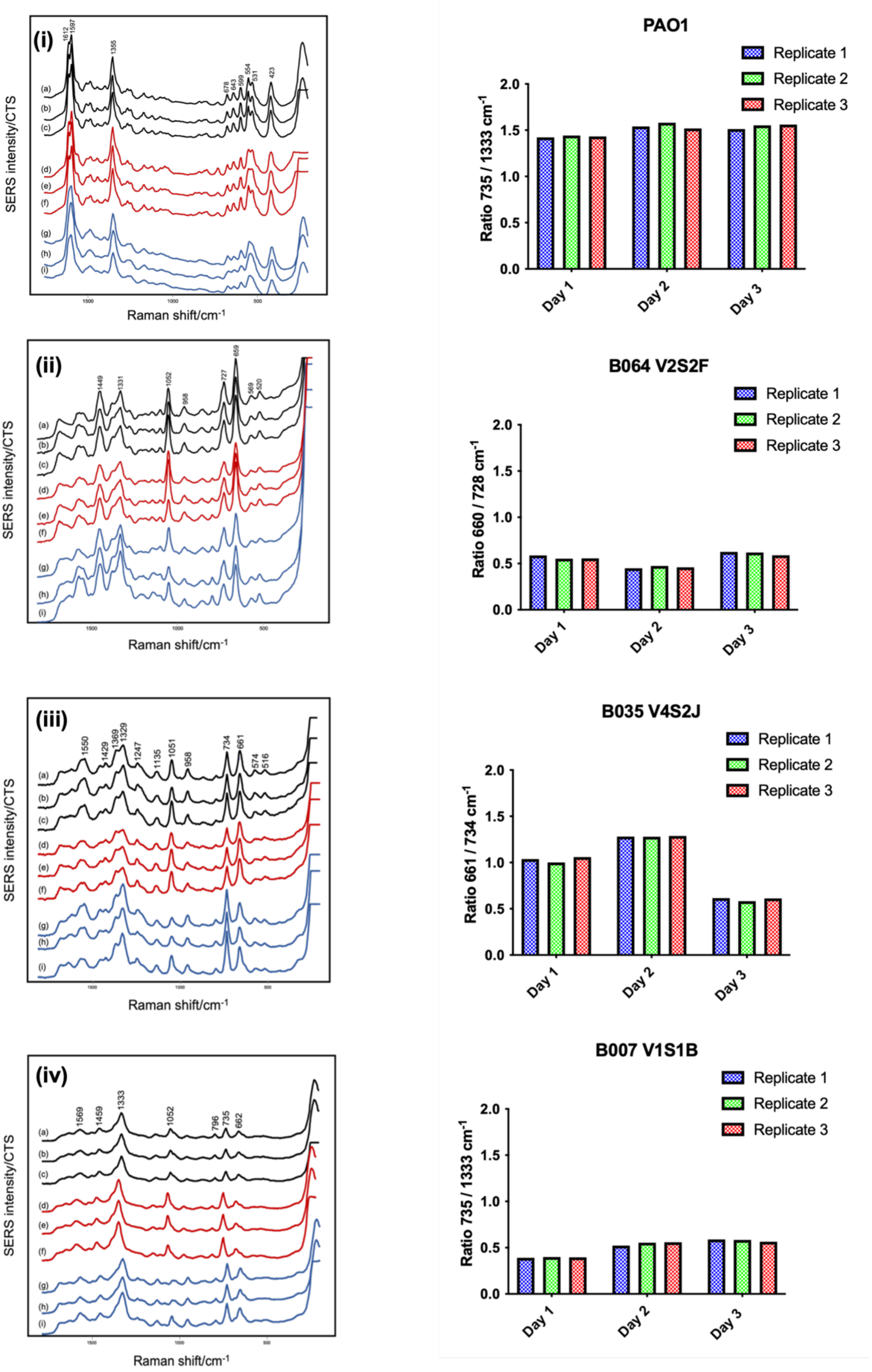

Figure S8. Illustration of the reproducibility of the SERS spectra of (i) *P. aeruginosa* PA01, (ii) *A. xylosoxidans* B064 V2S2F, (iii) *S. maltophilia* B035 V2S2J, (iv) *B. multivorans* B007 V1S1B shown as 3 technical replicates recorded on 3 different days: (a-c) Day 1, (d-f) Day 2 and (g-i) Day 3 and comparison of the ratio of the relative intensities of two vibrational bands.

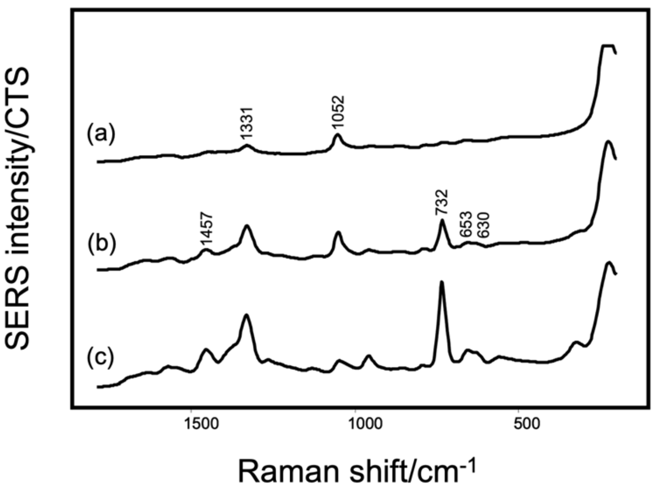

Figure S9: Comparison of the SERS spectra of *S. aureus* 15A at 3 different bacterial densities ~(a)4.7x10^{7} (b)2x10^{8} (c)7.3x10^{8} CFU/mL. Spectra shown are averages of data acquired in triplicate and repeated on 3 days. Spectra shown are offset but with same intensity scale.

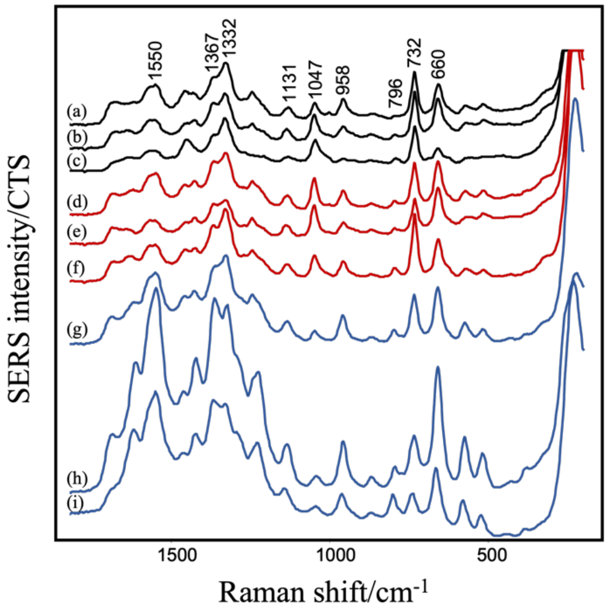

Figure S10. Data showing the effect growth phase on SERS spectra of *S. maltophilia* B035 V4S2J at (a-c) 3 hours (d-e) 6 hours and (f-g) 24 hours on Day 1, 2 and 3, respectively. Three technical replicates were acquired from the same sample and an average of the 3 spectra is presented.

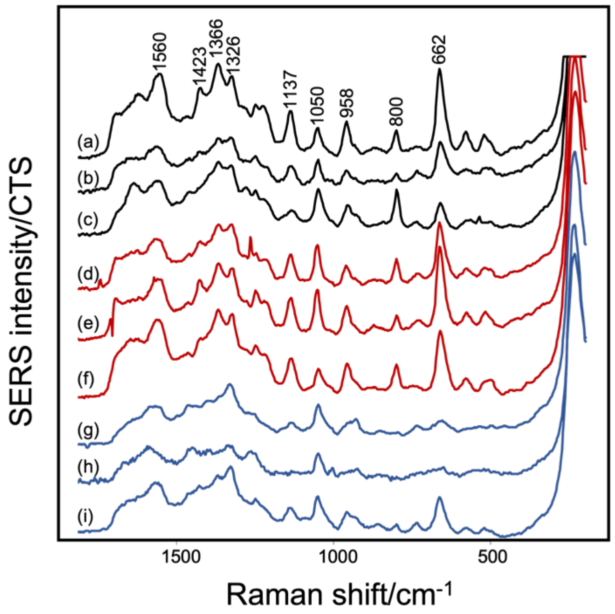

Figure S11. Data showing the effect growth phase on SERS spectra of *P. aeruginosa* AUS 454 at (a-c) 3 hours (d-e) 6 hours and (f-g) 24 hours on Day 1, 2 and 3, respectively. Three technical replicates were acquired from the same sample and an average of the 3 spectra is presented.

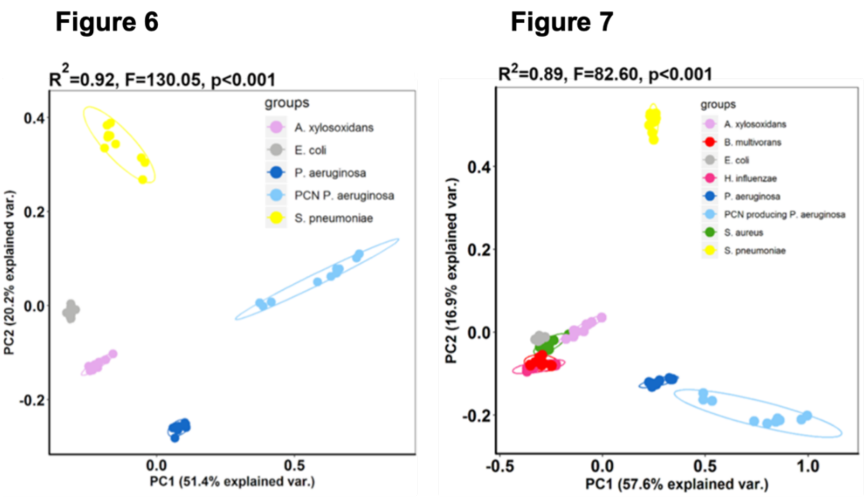

Figure S12. PCA plots annotated with the results from the PERMANOVA analysis

The differences in centroids between groups were compared by PERMANOVA, this indicated that there was significant separation between the means of the groups in both PCA plots (Figure 6 (R^2 = 0.92, F = 130.05, p<0.001) and Figure 7 (R^2 = 0.89, F = 82.60, p<0.001)). However, while this may indicate that there was significant separation between the means of the groups, it is clear from visual inspection of the PCA plot (Figure 7) that there was overlap between species and therefore, more challenging to differentiate. Further PCs were analyzed in Figure S13 and S14.

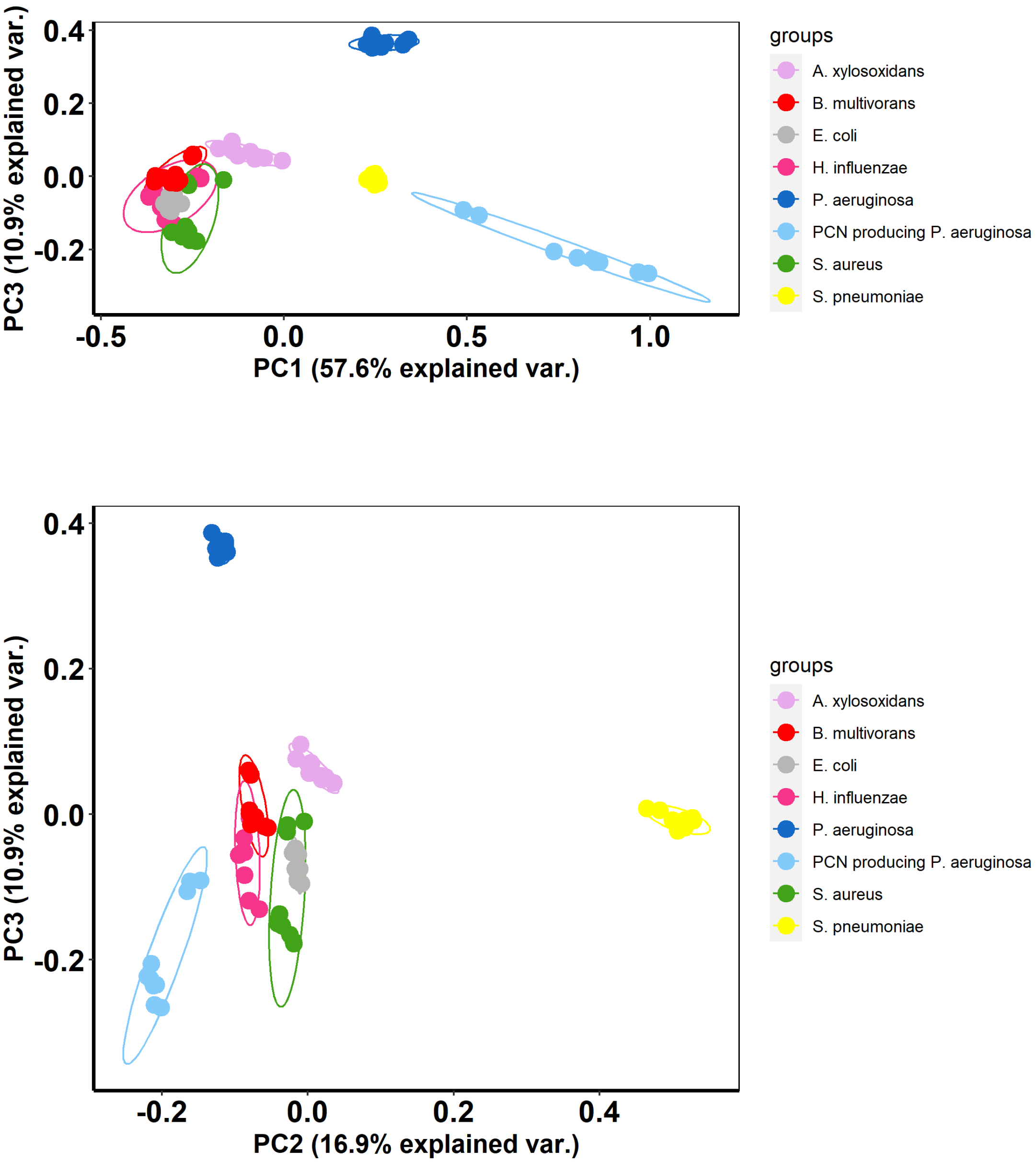

Figure S13. PCA plot illustrating the relationship between the SERS spectra of 8 bacterial isolates with 2 principal components (PC) (i) PC1 *v* PC3, accounting for 57.6% and 10.9%, total variance explained, respectively and (ii) PC2 v PC3, accounting for 16.9% and 10.9%, total variance explained, respectively.

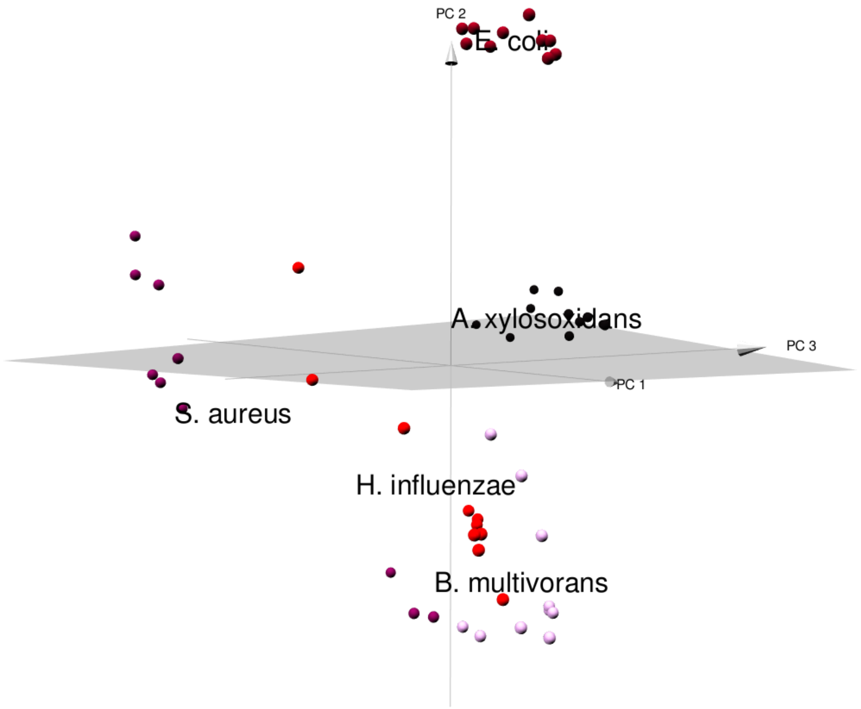

Figure S14. PCA plot illustrating the relationship between the SERS spectra of 5 bacterial species with three principal components. PC1, PC2 and PC3 accounted for 57.6%, 16.9% and 10.9% of the total variance explained, respectively. The main defined clusters were removed from this analysis to investigate more closely the bacterial species that overlapped on the PCA plot in Figure S13.
